# Phytochemical Analysis and Therapeutic Potential of *Tuberaria lignosa* (Sweet) Samp. Aqueous Extract in Skin Injuries

**DOI:** 10.3390/plants14152299

**Published:** 2025-07-25

**Authors:** Manuel González-Vázquez, Ana Quílez Guerrero, Mónica Zuzarte, Lígia Salgueiro, Jorge Alves-Silva, María Luisa González-Rodríguez, Rocío De la Puerta

**Affiliations:** 1 Department of Pharmacology, Faculty of Pharmacy, University of Seville, 41012 Seville, Spain; mgonzalez15@us.es (M.G.-V.); quilez@us.es (A.Q.G.); 2 Faculty of Pharmacy, University of Coimbra, Azinhaga de S. Comba, 3000-548 Coimbra, Portugal; mzuzarte@uc.pt (M.Z.); ligia@ff.uc.pt (L.S.); jmasilva@ff.uc.pt (J.A.-S.); 3 Faculty of Medicine, Coimbra Institute for Clinical and Biomedical Research, Centre for Innovative Biomedicine and Biotechnology (iCBR-CIBB), University of Coimbra, Azinhaga de S. Comba, 3000-548 Coimbra, Portugal; 4 Department of Chemical Engineering, Chemical Engineering and Renewable Resources for Sustainability (CERES), University of Coimbra, 3030-790 Coimbra, Portugal; 5 Department of Pharmaceutical Technology, Faculty of Pharmacy, University of Seville, 41012 Seville, Spain; malugoro@us.es

**Keywords:** *Tuberaria lignosa*, phenolic compounds, punicalagin, aqueous extract, antioxidant, wound healing, extracellular matrix enzymes, antifungal, antibiofilm

## Abstract

*Tuberaria lignosa* (Sweet) Samp. (Cistaceae) is a herbaceous species native to southwestern Europe, traditionally used to treat wounds, ulcers, and inflammatory or infectious skin conditions. This study aimed to characterize the phytochemical profile of its aqueous leaf extract and evaluate its skin-related in vitro biological activities. The phenolic composition was determined using UHPLC-HRMS/MS, HPLC-DAD, and quantitative colorimetric assays. Antioxidant activity was assessed against synthetic free radicals, reactive oxygen and nitrogen species, transition metals, and pro-oxidant enzymes. Enzymatic inhibition of tyrosinase, hyaluronidase, collagenase, and elastase were evaluated using in vitro assays. Cytocompatibility was tested on human keratinocytes and NIH/3T3 fibroblasts using MTT and resazurin assays, respectively, while wound healing was evaluated on NIH/3T3 fibroblasts using the scratch assay. Antifungal activity was investigated against several *Candida* and dermatophyte species, while antibiofilm activity was tested against *Epidermophyton floccosum*. The extract was found to be rich in phenolic compounds, accounting for nearly 45% of its dry weight. These included flavonoids, phenolic acids, and proanthocyanidins, with ellagitannins (punicalagin) being the predominant group. The extract demonstrated potent antioxidant, anti-tyrosinase, anti-collagenase, anti-elastase, and antidermatophytic activities, including fungistatic, fungicidal, and antibiofilm effects. These findings highlight the potential of *T. lignosa* as a valuable and underexplored source of bioactive phenolic compounds with strong potential for the development of innovative approaches for skin care and therapy.

## 1. Introduction

The Wound Healing Society defines wounds as physical injuries caused by a break or opening in the skin, which disrupt the structure and normal function of the skin [1]. Lesions can be categorized according to their cause, infection status, tissue damage, and healing progression. Acute wounds usually heal within four weeks through a process involving hemostasis, inflammation, proliferation, and remodeling [2]. However, this process may become dysregulated, especially in infected wounds or patients with comorbidities like diabetes, obesity, vascular diseases, or cancer [3]. Chronic wounds, which affect 2–6% of the global population, are associated with high morbidity and mortality rates, and have an impact on health-related quality of life comparable to that of cardiovascular and respiratory diseases [4,5]. From a pathophysiological perspective, factors such as ischemia, infections, and sustained growth factor activity can promote chronic inflammation via pro-inflammatory cytokines (e.g., IL-1, IL-6, TNF-α), matrix metalloproteinases (MMPs), and other extracellular matrix (ECM) degrading enzymes as well as reactive oxygen and nitrogen species (ROS and RNS, respectively). This can lead to ECM degradation, impaired cell migration, and a self-perpetuating inflammatory cycle [6]. Effective treatment includes controlling inflammation, removing necrotic tissue, managing exudate, promoting angiogenesis, and controlling infection [7].

In this sense, controlling infection and biofilms is pivotal to managing chronic wounds [8]. Biofilms, which are microbial communities in a matrix of polysaccharides and proteins, enhance pathogen resistance and perpetuate inflammation [9]. Advances in microbiological techniques have increased our understanding of the wound microbiome. Although *Staphylococcus*, *Pseudomonas*, and *Streptococcus* are the most common pathogens, fungal infections are increasingly recognized, especially in cases of diabetic foot ulcers and burns. Fungal pathogens are identified in 25–40% of chronic wounds using standard methods, and up to 80% of cases via molecular diagnostics [10,11]. *Candida*, *Aspergillus*, and dermatophyte fungi (mainly *Trichophyton*, *Microsporum*, and *Epidermophyton*) are particularly implicated [12]. In general, besides their importance in wounds, these fungal infections can cause significant morbidity and may disseminate in immunocompromised patients [13,14]. *Candida* spp. and dermatophytes affect millions of people annually, resulting in a substantial economic burden [15,16]. Treatment options include allylamines, azoles, and polyenes, while echinocandins are used for severe candidiasis [17,18]. However, concerns have been raised regarding systemic toxicity and increasing antifungal resistance (e.g., *T. indotineae*) [15]. In response, research on plant-derived phenolic compounds, such as tannins, flavonoids, and phenolic acids, has been intensified. These compounds have shown a great potential in modulating inflammation, oxidative stress, ECM degradation, and microbial infections, and are being developed for use in modern clinical formulations for chronic lesions and recurrent skin infections [19].

*Tuberaria lignosa* (Sweet) Samp., a rockrose-like species from the *Cistaceae* family, is native to western and southern Europe, with a particularly strong presence in the Iberian Peninsula. In Spain, it is commonly known as chaguarcina, jaracepa, or zaragocilla, while in Portugal, it is referred to as alcária or erva-loba [20]. Traditionally, this plant has been wild harvested to prepare decoctions and infusions for domestic use, primarily in folk medicine in the Iberian Peninsula, to treat various ailments in both humans and domestic animals, such as skin infections, wounds, and burns as well as in gastrointestinal issues [21,22]. In recent years, the limited research conducted on *T. lignosa* preparations has suggested the presence of potentially valuable polyphenolic compounds and has provided preliminary evidence of its anti-HIV, antitumor, and gastrointestinal properties [23,24,25,26].

However, the potential of this medicinal plant in skin-related conditions has yet to be explored. Based on its traditional uses and on the above-mentioned scientific evidence, we hypothesize that the aqueous leaf extract of *T. lignosa* (TLAE) has important antioxidant, skin health-related enzyme inhibitory, wound healing, and antifungal properties. Therefore, the purpose of this study is to validate the traditional use of *T. lignosa* in the treatment of skin injuries, such as wounds and fungal infections, and to establish its relevance as a bioactive agent in skin health. To this end, this study aims to characterize the phytochemical profile of TLAE to identify its bioactive compounds, and to evaluate its antioxidant, skin health-related enzyme inhibitory, wound-healing, and antifungal activities through diverse in vitro experimental models.

## 2. Results

### 2.1. Phytochemical Characterization of Tuberaria lignosa (Sweet.) Samp. Aqueous Leaf Extract

#### 2.1.1. Extraction Yield

Extracting *T. lignosa* dried leaves (15.5 g) with boiling distilled water (500 mL) resulted in an average extract weight of 4.39 ± 0.53 g, corresponding to an extraction yield of 28.29 ± 3.40% (*w*/*w*). This is expressed as the mean ± standard deviation (SD) from three independent extractions.

#### 2.1.2. Quantification of Phenolic Constituents Through Colorimetric Assays

The phytochemical composition of TLAE, summarized in Table 1, revealed a total phenolic content of 425.33 ± 11.02 mg of Gallic acid equivalents (GAE) per gram of dry extract. This was followed by a total flavonoid content of 181.21 ± 6.04 mg of Rutin equivalents (RE) per gram of dry extract, and a total hydroxycinnamic acid content of 28.88 ± 3.08 mg of Caffeic acid equivalents (CAE) per gram of dry extract. Proanthocyanidins were detected at 38.64 ± 4.01 mg of Catechin equivalents per gram of dry extract, while total tannins accounted for 71.96 ± 2.25% *w*/*w* expressed as Tannin-related phenolics (TRP).

#### 2.1.3. UHPLC-HRMS/MS and HPLC-DAD Phenolic Profile Analysis

Phytochemical analysis by UHPLC-HRMS/MS revealed the presence of multiple phenolic compounds, and organic acids, including quinic acid, as well as the phytohormone abscisic acid. Twenty phenolic compounds were identified, including four hydroxybenzoic acids (gallic acid, protocatechuic acid, salicylic acid, and ellagic acid); two hydroxycinnamic acids (caffeic acid and p-coumaric acid); two flavan-3-ols ((-)-gallocatechin and catechin); one procyanidin (procyanidin B1); one ellagitannin (punicalagin); two dihydroflavonols (taxifolin and aromadendrin), one flavanone (naringenin), two flavonols (morin and kaempferol), two O-glycosylated flavonols (hyperoside and kaempferol-3,7-O-α-diramnopyranoside), two C-glycosylated flavones (vitexin and isovitexin), and one phenolic aldehyde (vanillin). Of the compounds identified and quantified, the flavones vitexin and isovitexin were the most abundant (Table 2).

Additionally, HPLC-DAD analysis of the punicalagin standard revealed a characteristic chromatographic profile with two distinct peaks, corresponding to mean ± SD retention times of 4.21 ± 0.10 min (tentatively assigned to the α-anomer) and 4.61 ± 0.24 min (tentatively assigned to the β-anomer), both showing maximum absorbance at 258/378 nm. In turn, the chromatogram of TLAE displayed two peaks with mean ± SD retention times of 4.24 ± 0.06 and 4.63 ± 0.11 min, also with maximum absorbance at 258/378 nm (see Supplementary Document S2 for representative chromatograms, shown as typical examples of both the standard and the TLAE analyses). Based on these findings, the punicalagin content in TLAE was quantified as 226.75 ± 13.90 mg/g of dry extract.

### 2.2. Evaluation of In Vitro Antioxidant Capacity of T. lignosa Aqueous Leaf Extract

#### 2.2.1. DPPH^•^ and ABTS^•+^ Free-Radical Scavenging Capacity

The scavenging capacity of TLAE against synthetic free-radicals was determined by measuring the 1,1-diphenyl-2-picrylhydrazyl (DPPH^•^) and 2,2′-azino-bis (3-ethylbenzothiazoline-6-sulfonic acid) diammonium salt (ABTS^•+^) radical cations (Table 3). The results indicate that TLAE exhibits a potent free radical scavenging capacity, with an IC_50_ similar to that of Trolox for both assays (9.65 ± 0.49 μg/mL and IC_50_ 6.25 ± 0.07 µg/mL, respectively). This is further supported by the Trolox Equivalent Antioxidant Capacity (TEAC) value (TEAC_TLAE_ = 1.08 ± 0.096) and the Antioxidant Activity Index (AAI), the latter categorizing TLAE as a very strong antioxidant (AAI_TLAE_ = 4.56 ± 0.21) according to Scherer and Godoy [27].

#### 2.2.2. Reactive Oxygen and Nitrogen Species Scavenging Capacity

The scavenging capacity of TLAE against reactive oxygen species (ROS) and reactive nitrogen species (RNS) was determined by measuring the H_2_O_2_, OH^•^, O_2_^−•^, and ^•^NO radicals in cell-free systems (Table 3). These results suggest that TLAE exhibits significant antioxidant activity against ROS and RNS. It has a similar H_2_O_2_ scavenging capacity (IC_50_ 9.43 ± 0.44 µg/mL) to trolox (IC_50_ 9.93 ± 0.45 µg/mL), and remarkable OH^•^, O_2_*^−^*^•^, and ^•^NO antioxidant capacities (OH^•^ IC_50_: 313.51 ± 24.82 µg/mL; O_2_*^−^*^•^ − IC_50_: 8.81 ± 0.71 µg/mL; ^•^NO IC_50_: 35.22 ± 1.33 µg/mL), compared to different reference standards.

#### 2.2.3. Transition Metal Chelating Capacity

The transition metal chelating capacity of TLAE was studied by the ferrous ion (Fe^2+^) uptake assay (Table 3). Our results indicate that TLAE (IC_50_ 10.54 ± 0.35 µg/mL) can effectively chelate transition metals such as Fe^2+^ in a similar extent as EDTA (IC_50_ 7.83 ± 0.30 µg/mL).

#### 2.2.4. Xanthine Oxidase Inhibition Capacity

**Figure 1:**
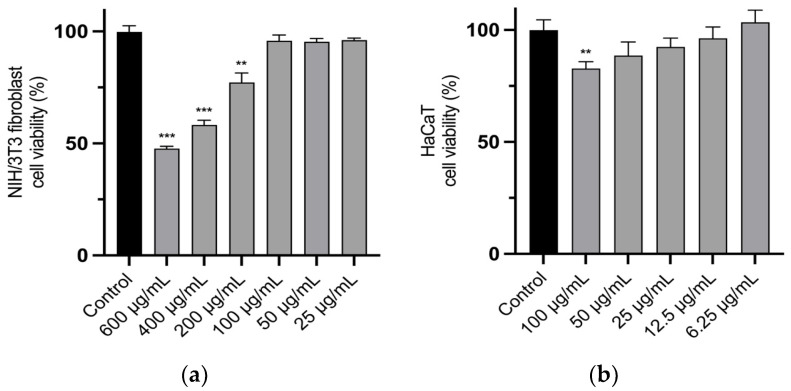
Effect of *Tuberaria lignosa* (Sweet) Samp. aqueous extract on cell viability of (**a**) NIH/3T3 fibroblasts (resazurin assay) and (**b**) HaCaT cells (MTT assay). Results are expressed as percentage of cell viability compared to control. Columns and bars represent mean and standard deviation, respectively (*n* = 4). Statistical analysis was performed by one-way ANOVA followed by Dunnett’s Multiple Comparison Test: ** *p* < 0.01, *** *p* < 0.001, compared to control.

The inhibitory activity of xanthine oxidase was evaluated using allopurinol as a reference standard, with uric acid production serving as the assay indicator (Table 3). Our results show that TLAE exhibited a promising inhibitory effect on this enzyme at relatively low and non-toxic doses (IC_50_ 27.6 ± 1.59 µg/mL) as it was tested in the culture cell lines (Figure 1).

### 2.3. Evaluation of Skin-Related Enzyme Inhibitory Activity of T. lignosa Aqueous Leaf Extract

The in vitro skin-related enzyme inhibitory activity of TLAE was evaluated in relation to collagenase, elastase, hyaluronidase, and tyrosinase by using epigallocatechin gallate (EGCG), quercetin, tannic acid, and kojic acid as reference standards, respectively (Table 4). Our results indicate that TLAE exhibited a very noteworthy activity as an elastase (IC_50_ 219.02 ± 11.3 µg/mL), tyrosinase (IC_50_ 67.48 ± 2.78 µg/mL), and collagenase (IC_50_ 58.49 ± 4.08% of inhibition at 200 µg/mL) inhibitor. Indeed, the TLAE inhibition values for tyrosinase and collagenase are similar to the values of the standards used. In contrast, the extract showed no activity against hyaluronidase at doses up to 675 µg/mL.

### 2.4. Evaluation of T. lignosa Aqueous Leaf Extract on Cell Viability in NIH/3T3 Fibroblast and HaCaT Cells

The effect of different concentrations of TLAE on the viability of NIH/3T3 fibroblasts (Figure 1a) and HaCaT cells (Figure 1b) was determined using the resazurin reduction assay and MTT assay, respectively. In general, TLAE showed no or slight toxicity on NIH/3T3 fibroblasts up to concentrations of 200 µg/mL compared to the control group according to ISO 10993-5:2009 [28]. Furthermore, according to the MTT assay, no or slight toxicity was observed in HaCaT cells up to 100 µg/mL compared to the control group.

### 2.5. Evaluation of T. lignosa Aqueous Leaf Extract on Cell Migration in NIH/3T3 Fibroblast

**Figure 2:**
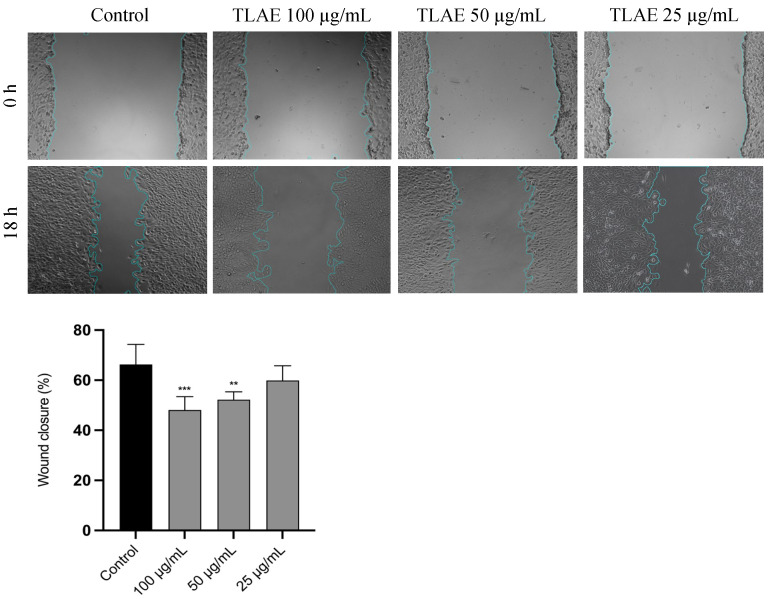
Evaluation of the wound healing effect of *Tuberaria lignosa* (Sweet) Samp. aqueous extract on NIH/3T3 fibroblasts (Scratch assay). Data are expressed as the comparison, as percentage of wound closure, between the wound area at 0 and 18 h. Columns and bars represent mean and standard deviation, respectively (*n* = 6). Statistical analysis was performed by one-way ANOVA followed by Dunnett’s Multiple Comparison Test: ** *p* < 0.01, *** *p* < 0.001, compared to control.

The effect of TLAE on the cell migration of NIH/3T3 fibroblasts was determined using the scratch wound assay. As shown in Figure 2, TLAE at 25 µg/mL produced a comparable percentage of wound closure area as control (*p* > 0.05). However, a dose-dependent inhibitory effect on fibroblast cell migration was observed at concentrations up to 100 µg/mL (*p* < 0.001).

### 2.6. Evaluation of the Antifungal Activity of T. lignosa Aqueous Leaf Extract

The antifungal potential of TLAE was evaluated against various yeast and dermatophyte strains, including clinical isolates, to assess its effectiveness against more resistant fungi (Table 5). TLAE demonstrated a particularly important antidermatophytic activity, with a minimum inhibitory concentration (MIC) of 50 µg/mL against *Trichophyton mentagrophytes* var. *interdigitale*, and 100 µg/mL against *Epidermophyton floccosum*, *Microsporum canis*, *T. mentagrophytes*, and *T. rubrum*. For *M. gypseum* a MIC value of 400 µg/mL was obtained. In addition, the extract exhibited fungistatic activity against *Candida krusei* (MIC = 1000 µg/mL). Notably, for most strains with positive antifungal activity, MIC and MLC values were similar, indicating a fungicidal effect rather than just fungistatic activity. No antifungal activity was observed against *T. verrucosum* and the remaining *Candida* species at concentrations up to 1000 µg/mL.

### 2.7. Evaluation of the Antibiofilm Activity Against Epidermophyton floccosum FF9 Biofilms of T. lignosa Aqueous Leaf Extract

#### 2.7.1. Effect on the Formation of *E. floccosum* Biofilms

**Figure 3:**
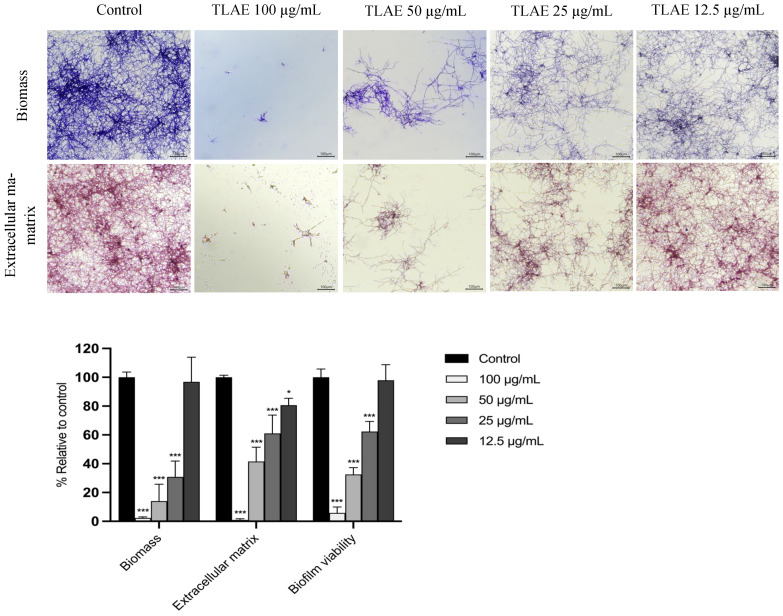
Effect of *Tuberaria lignosa* (Sweet) Samp. aqueous leaf extract on *Epidermophyton floccosum* FF9 biofilm formation. Results are expressed as a percentage relative to control for biomass, extracellular matrix, and biofilm viability. Columns and bars represent mean and standard deviation, respectively (*n* = 3 performed in duplicate). Statistical analysis was performed by two-way ANOVA followed by Dunnett’s Multiple Comparison Test: * *p* < 0.05, *** *p* < 0.001, compared to control. Scale bar = 100 µm.

*E. floccosum* was selected to evaluate the antibiofilm activity of TLAE due to the limited information available on this common dermatophyte fungus, which is capable of forming biofilms in vitro and in clinical settings such as superficial infections, chronic wounds, and deep soft tissue infections [29,30]. TLAE was found to significantly inhibit biofilm biomass, extracellular matrix production, and biofilm viability compared to the control group (*p* < 0.001) within a concentration range of 25 µg/mL to 100 µg/mL (Figure 3).

#### 2.7.2. Effect on the Disruption of *E. floccosum* Mature Biofilms

**Figure 4:**
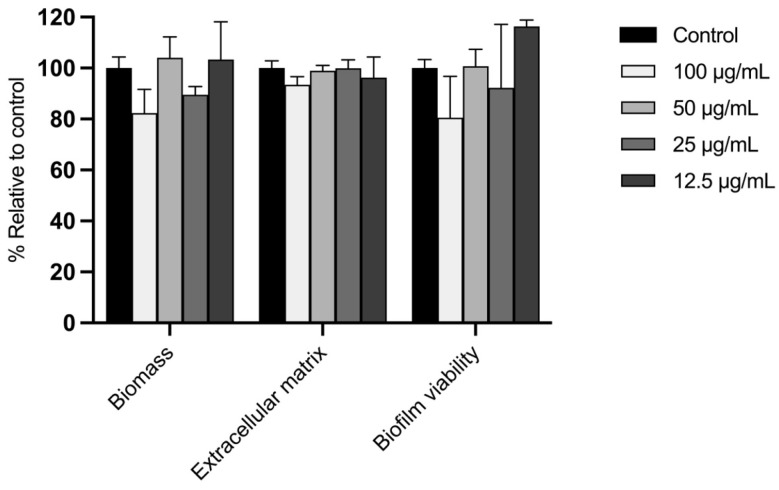
Effect of *Tuberaria lignosa* (Sweet) Samp. aqueous leaf extract on *Epidermophyton floccosum* FF9 mature biofilm disruption. Results are expressed as a percentage relative to control for biomass, extracellular matrix, and biofilm viability. Columns and bars represent mean and standard deviation, respectively (*n* = 3 performed in duplicate). Statistical analysis was performed by two-way ANOVA followed by Dunnett’s Multiple Comparison Test.

Considering the resistance of mature biofilms to antifungal agents, we evaluated the ability of TLAE to disrupt these structures in *E. floccosum* biofilms (Figure 4). As expected, mature biofilms were more resistant to the activity of the extract, showing no reduction in biofilm biomass, extracellular matrix formation, or biofilm viability compared to the control group at any of the tested concentrations (*p* > 0.05).

## 3. Discussion

*Tuberaria lignosa* (Sweet) Samp. (Cistaceae) is a herbaceous species distributed across western and southern Europe, particularly in the western Iberian Peninsula. It has traditionally been used to treat wounds and other inflammatory or skin infectious and digestive conditions [20,21,22]. Despite this ethnopharmacological relevance, scientific studies on its bioactive potential are scarce, with no reports specifically exploring its dermatological applications [23,24]. In this context, the present study aims to characterize the phytochemical profile of *T. lignosa* leaf aqueous extract (TLAE) and evaluate its bioactivity in skin-related conditions. The extract showed a phenolic-rich composition, mainly consisting of ellagitannins and glycosylated flavones, which may explain its potent antioxidant activity against synthetic radicals, ROS, RNS, transition metals, and xanthine oxidase. The extract also significantly inhibited tyrosinase, elastase, and collagenase, enzymes linked to skin ageing and tissue remodeling, and exhibited fungistatic, fungicidal, and antibiofilm effects against several dermatophytes. Most effects occurred at concentrations that were non-toxic for NIH/3T3 fibroblasts or HaCaT cells. Additionally, in vitro wound healing assay revealed wound closure similar to control at 25 µg/mL and a dose-dependent inhibition of NIH/3T3 fibroblast migration at doses of 50 µg/mL and 100 µg/mL.

Polyphenols are widespread in the plant kingdom and are known for their important preventive and therapeutic properties [31]. In this context, TLAE can be considered as a polyphenol-rich extract, with nearly 45% total phenolic content (*w/w*) according to the Folin–Ciocalteu assay, surpassing values reported for other species from Cistaceae family [32,33]. Most of the phenolic compounds appear to be hydrolyzable tannins, as suggested by total tannin and proanthocyanidin quantification assays. UHPLC-HRMS/MS and HPLC-DAD analyses confirmed these results, revealing over 20 phenolic compounds, including several that were reported for the first time in this species: hydroxybenzoic acids (gallic, protocatechuic, and salicylic acids), hydroxycinnamic acids (caffeic and p-coumaric acids), flavan-3-ols (catechin and (-) gallocatechin), procyanidins (procyanidin B1), dihydroflavonols (taxifolin and aromadendrin), flavanones (naringenin), flavonols (morin), O-glycosylated flavonols (hyperoside and kaempferol-3,7-O-α-dirhamnopyranoside) as well as phenolic aldehydes (vanillin). Considerable levels of vitexin and isovitexin were also detected (216.1 ± 2.54 µg/g and 375.44 ± 7.49 µg/g of dry extract, respectively). This profile enriches previous reports on *T. lignosa* and aligns for other species from Cistaceae family [25,34], notably for its high punicalagin content (>20% *w/w* dry extract), which exceeds that of related taxa [35,36]. This high phenolic content may reflect species-specific antioxidant responses to Mediterranean conditions, as observed in *T. globulariifolia* which, alongside *T. lignosa*, is one of only two non-therophyte species in the genus [37,38].

Punicalagin (C_48_H_28_O_30_) is a high-molecular-weight ellagitannin (1084.71 g/mol) that is present in the α- and β-isomeric forms in plants belonging to the families Combretaceae, Lythraceae and Cistaceae. Its multiple hydroxyl groups contribute to radical scavenging, metal chelation, and modulation of multiple antioxidant pathways [39]. This has also been observed for flavone C-glycosides, such as vitexin and isovitexin, which exhibit a broad range of biological activities relevant to skin health [40]. In order to evaluate the potential of TLAE in wound healing, fungal infections, and skin health, its response to oxidative stress should also be taken into account. Although oxidative stress, caused by excess ROS, RNS, Fe^2+^, and enzymes such as XO has important physiological roles, an imbalance can leads to tissue damage, impaired healing, exacerbated infection, and accelerated aging [41]. Antioxidants mitigate this damage by preventing oxidation at low concentrations [42], with efficacy often being linked to phenolic content and hydroxyl group structure [43]. In this study, TLAE exhibited strong antioxidant activity in conventional DPPH^•^ and ABTS^•+^ assays, as well as, for the first time, in physiologically relevant models targeting ROS, RNS, transition metals, and XO. It’s DPPH^•^-IC_50_ value classifies TLAE as a “very strong antioxidant” [27], which is consistent with previous findings related to the Cistaceae family [32,44]. TLAE also exhibited potent Fe^2+^ chelating activity and radical scavenging capacity against H_2_O_2_, O_2_^−•^, and ^•^NO, which are some of the most toxic pro-oxidant agents in biological systems [45]. Furthermore, TLAE inhibited XO, an enzyme producing O_2_^−•^ during purine metabolism and implicated in chronic wound pathogenesis [46]. This high antioxidant activity is probably attributable to both the elevated content, particularly of punicalagin, and the specific nature of phenolic compounds in TLAE [47].

Although the antioxidant capacity of punicalagin and punicalagin-rich extracts is well established, their effects on cellular systems can vary depending on the concentration and the experimental conditions [39]. TLAE showed negligible cytotoxicity in NIH/3T3 fibroblasts at concentrations up to 200 µg/mL and in HaCaT cells up to 100 µg/mL, according to ISO 10993-5:2009 [28]. In this context, the dose-dependent inhibition of NIH/3T3 fibroblast migration by TLAE, in the scratch assay, may be related to a hormetic response triggered by certain polyphenols, including punicalagin [48,49]. This hypothesis is strongly supported by various studies indicating that high concentrations of punicalagin or punicalagin-rich extracts, similar to those employed in our study, can impair the migration of fibroblasts and HaCaT cells through different mechanisms, including the AMPK, PI3K/Akt, and NF-κB signaling pathways, which regulate processes such as apoptosis, cell cycle arrest, autophagy, and mitochondrial dysfunction, while lower doses (~10^−6^ M) may have a stimulatory effect [26,50,51,52,53,54]. Nevertheless, in vivo and ex vivo studies show that punicalagin-rich formulations support wound healing, through angiogenic, antimicrobial, and immunomodulatory effects. This suggests that responses in more complex models may differ fundamentally from those in monolayer cell cultures [55,56,57]. Similarly, the C-glycosylated flavones vitexin and isovitexin promoted migration of NIH/3T3 fibroblasts more effectively at lower doses [58]. However, this effect may be masked by the considerably higher proportion of punicalagin in the extract.

During skin pathophysiological processes where inflammation and oxidative stress predominate, triggered by UV exposure, infections, pollution, aging, or comorbidities, stromal and immune cells release enzymes that degrade the extracellular matrix (ECM), such as matrix metalloproteinases (e.g., collagenases), serine proteases (e.g., neutrophil elastase), and hyaluronidases. Chronic overexpression of these enzymes can degrade key skin macromolecules, thereby impairing tensile strength, hydration, and the healing process. This contributes to the atrophy and wrinkling of aged skin and delaying wound healing in chronic conditions [59,60]. TLAE was found to effectively inhibit collagenase, with comparable efficacy to EGCG, and elastase. This underscores its promising potential as a dual selective enzymatic inhibitor and is noteworthy given the current lack of selective and safe inhibitors that are commercially available for this class of enzymes [61]. Additionally, ageing-related factors induces the overexpression of tyrosinase, which is a key enzyme in melanogenesis and pigmentary disorders such as melasma and senile lentigines. In this sense, TLAE was found to inhibit tyrosinase activity at non-toxic concentrations across all tested cell lines, suggesting its potential application in depigmentation therapies, particularly when considering the side effects associated with current agents such as hydroquinone and kojic acid [62]. To the best of our knowledge, this is the first study to evaluate the inhibitory activity of a species from the *Tuberaria* genus on key skin health-related enzymes, supporting the scarce evidence on the potential of species from the Cistaceae family as a source of collagenase, elastase, and tyrosinase inhibitors [63,64]. This is also consistent with previous studies on the inhibitory activity of punicalagin and other TLAE-related phenolic compounds, such as glycosylated flavones, on these enzymes [65,66,67,68,69].

In addition to the aforementioned pathological processes, fungal pathogens, particularly dermatophytes and *Candida* spp., are the leading cause of skin and mucosal infections worldwide. There is an increased risk in immunocompromised patients due to antifungal resistance and systemic complications, which are likely linked to biofilm formation [13,15]. In this context, the antifungal activity of TLAE against several dermatophytes and *Candida* strains, as well as its antibiofilm activity against *Epidermophyton floccosum*, was investigated. TLAE exhibited a strong antidermatophytic activity with MIC and MLC values below the cytotoxic thresholds for fibroblasts and keratinocytes, and fungistatic activity against *C. krusei* at higher concentrations. This antifungal activity may likely results from synergistic effects among phenolic compounds, including tannins, flavonoids, and phenolic acids, which are known to disrupt fungal membranes, ergosterol biosynthesis, and mitochondrial function [70,71,72]. Punicalagin, a compound with well-documented antifungal properties, is likely a key contributor to this effect [73]. In contrast, although no data are currently available regarding the antifungal activity of the C-glycosylated flavones vitexin and isovitexin against dermatophytes, these compounds have not shown significant antifungal effects against phytopathogenic filamentous fungi [74]. Moreover, TLAE exhibited an outstanding dose-dependent inhibition during early *E. floccosum* biofilm development, reducing biofilm biomass, ECM deposition, and fungal viability at subinhibitory concentrations as low as 25 µg/mL. However, no activity was observed on mature biofilms, likely because, once biofilms are established, their compact ECM can act as a multifaceted barrier that limits drug diffusion and interferes with phenolic compounds through different mechanisms [75,76]. Beyond the intrinsic value of advancing antifungal research, all these promising findings are particularly significant as they are likely to be the first on a *Tuberaria*-derived extract. These results also contribute to the limited antifungal research on non-*Cistus* species from Cistaceae family, highlighting the strong potential of *Tuberaria lignosa* as a source of future treatments for dermatophytosis.

Finally, it should be noted that certain aspects of this study warrant further investigation. Indeed, more plant samples should be tested to assess putative chemical variability. To further confirm our promising in *chemico* results, cell-based systems should be used and, regarding antidermatophytic activity of TLAE, additional studies underlying the mechanisms of both the aqueous extract of *T. lignosa* and its major compounds, punicalagin, isovitexin, and vitexin, should be considered.

## 4. Materials and Methods

### 4.1. Chemicals, Reagents and Equipment

Most reagents were acquired from Sigma Aldrich Co. (St. Louis, MO, USA). Punicalagin standard was purchased from Chengdu Biopurify Phytochemicals Ltd. (Chengdu, China). 1,1-diphenyl-2-picrylhydrazyl (DPPH), allopurinol, and ferrozine were purchased from TCI Europe N.V. (Zwijndrecht, Belgium). Taxifolin, vitexin, isovitexin, hyperoside, caffeic acid, and (+)-catechin was purchased from Extrasynthese (Genay, France). Vanillin and rutin were purchased from Merck KGaA (Darmstadt, Germany). Folin and Ciocalteu′s phenol reagent was purchased from Panreac AppliChem ITW Reagents S.R.L (Monza, Italy). Aluminum trichloride hexahydrate was purchased from Acofarma (Barcelona, Spain). 1-vinyl-2-pyrrolidinone polymer (PVPP) was purchased from Indagoochem (Barcelona, Spain). Kaempferol-3,7-di-O-rhamnoside was kindly supply by Prof. Martín-Cordero C. (Department of Pharmacology, University of Seville, Seville, Spain). All solvents were purchased from VWR International S.A.S (Rosny-sous-Bois, France).

Unless otherwise specified, absorbance measurements for all assays were performed using a microplate spectrophotometer (iMark™ Microplate Reader, BIO-RAD, Hercules, CA, USA) at the appropriate wavelength for each specific assay.

Detailed information on the tests performed and the methodology used in this article is available as Supplementary Document S1 in the Supplementary Materials.

### 4.2. Plant Material

**Figure 5:**
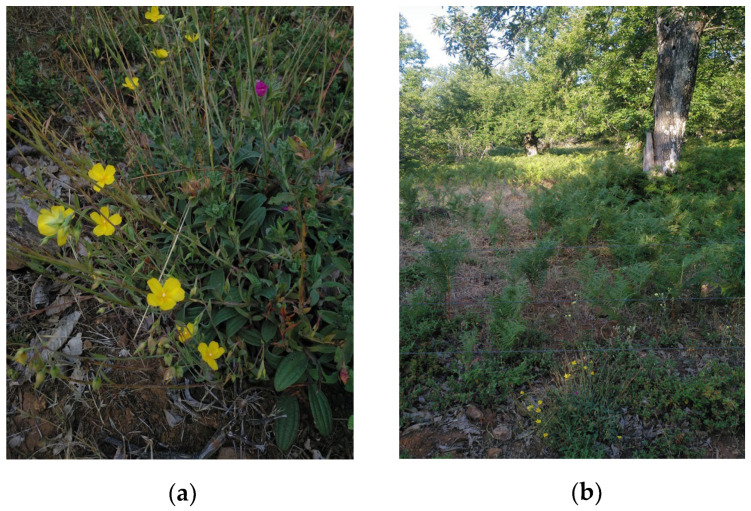
Typical flowering pattern of *Tuberaria lignosa* (Sweet) Samp. specimens (**a**), and their collection site, characterized by the presence of other species of rockroses, chestnut trees, and ferns (**b**). Images were captured by the authors.

*Tuberaria lignosa* (Sweet) Samp. leaves were collected during the flowering stage in late June 2023 in the environment of the Sierra de Aracena y Picos de Aroche Natural Park (Huelva, Spain) at GPS coordinates 37°53′03.3″N/6°47′08.1″W at 761 m a.s.l. (Figure 5). The harvest coincided with a period with significant thermometric and pluviometric anomalies (increased temperature and drought) above the historical average for that time according to data provided by the regional meteorological station (IFAPA, 2024) [77]. After identification and authentication by Prof. García-Murillo P. from the Department of Plant Biology and Ecology (University of Seville), a sample specimen was deposited in the Herbarium of the University of Seville (sample number: SEV 289985). The plant material reserved for experimentation was washed with distilled water and left to dry in the shade with mechanical ventilation for two weeks at a temperature of 29 °C and a relative humidity of 39%. The leaves were then ground to a size of between 5 and 10 mm. Finally, this material was stored under vacuum in polyethylene bags at a temperature of −20 °C until their subsequent use.

### 4.3. Extraction Procedure

The dried leaves (15.5 g) of *T. lignosa* were extracted with distilled water (500 mL) at boiling point for 5 min. After leaving the mixture at room temperature for 15 min, it was filtered using Whatman^®^ Grade 1 paper. Finally, the filtered extract was concentrated under reduced pressure, lyophilized and stored at −20 °C until further use.

### 4.4. Phytochemical Characterization

#### 4.4.1. Total Phenolic Content

Total phenolic content was determined by the Folin–Ciocalteu method [78], adapted to a microplate reader [79]. Gallic acid was used as a reference standard. Absorbance was measured at 750 nm, and results were expressed as mg of gallic acid equivalents (GAE) per g of dry extract.

#### 4.4.2. Total Flavonoid Content

Total flavonoid content was determined by the Lamaison–Carnat method adapted to a microplate reader [80]. Briefly, 100 µL of sample was mixed with 100 µL of aluminum trichloride (2% w/v in 96° ethanol) in a microtiter plate and, after 10 min, absorbance was measured at 405 nm. Rutin was used as a reference standard. Results were expressed as mg rutin equivalents (RE) per g dry extract.

#### 4.4.3. Total Hydroxycinnamic Acids Content

Total hydroxycinnamic acids content was determined by the method described by Arnow [81], adapted to a microplate reader [82]. Caffeic acid was used as a reference standard. Absorbance was measured at 490 nm, and results were expressed as mg of caffeic acid equivalents (CAE) per g of dry extract.

#### 4.4.4. Total Proanthocyanidin Content

Total proanthocyanidin content was determined following the recommendations of [83] adapted to a microplate reader [84]. (+)-Catechin was used as a reference standard. Absorbance was measured at 490 nm, and results were expressed as mg of catechin equivalents (CE) per g of dry extract.

#### 4.4.5. Total Tannin Content

Total tannin content was determined by the method described by [85] adapted to a microplate reader according to [86]. Tannic acid was used as a reference standard. Absorbance was measured at 750 nm, and results were expressed as % *w*/*w* of tannin-related phenolics relative to the total phenolic content (TRP) determined by the Folin–Ciocalteu assay.

#### 4.4.6. UHPLC-HRMS/MS and HPLC-DAD Phenolic Profile Analysis

Chromatographic separation was carried out on a UHPLC Dionex Ultimate 3000 RS system (Thermo Fisher Scientific, San Jose, CA, USA) equipped with a binary pump, an autosampler, and a column oven. An Acquity BEH C18 reversed-phase column (particle size 1.7 µm, 2.1 × 100 mm) provided by Waters (Milford, MA, USA) was used for the proposed method. Separation under gradient elution based on 0.1% formic acid aqueous solution (solvent A) and methanol also containing 0.1% formic acid (solvent B) was as follows: 0−1 min, isocratic conditions at 5% B; 1−10 min, linear gradient from 5% to 100%B; 10−12 min, isocratic step at 100% B; and finally, 12–15 min back to initial conditions at 5%B to re-equilibrate the column. The mobile phase flow rate was 0.5 mL min^−1^ and the injection volume employed was 5 μL. The UHPLC system was coupled to a Q-Exactive Orbitrap HRMS system (Thermo Fisher Scientific) equipped with a heated electrospray ionization source (HESI-II) operated in negative ionization mode. Nitrogen was used as a sheath gas, sweep gas, and auxiliary gas at flow rates of 60, 0, and 25 au (arbitrary units), respectively. HESI-II heater temperature at 400 °C and capillary voltage at −3.0 kV were applied. Instrument capillary temperature was set at 320 °C, and an SLens RF level of 50 V was used. The Q-Exactive Orbitrap HRMS system was tuned and calibrated using commercially available Thermo Fisher calibration solution every 7 days. The HRMS instrument was operated in full MS scan mode with a *m*/*z* range from 50 to 750 at a mass resolution of 70,000 full width at half-maximum (FWHM) at *m*/*z* 200, with an automatic gain control (AGC) target (the number of ions to fill the C-Trap) of 3.0E6 with a maximum injection time (IT) of 200 ms. Full MS scan mode was followed by a data-dependent scan operated product ion scan mode (Top5) and applying for the fragmentation stepped normalized collision energies (NCE) of 30, 60, and 90 eV. Product ion spectra with an isolation window of 0.7 *m*/*z* was used. At this stage, a mass resolution of 17,500 FWHM at *m*/*z* 200, with an AGC target at 2.0 × 10^5^, and a maximum IT of 50 ms were employed. Data-dependent scan was triggered with an intensity threshold of 1.6 × 10^5^. Xcalibur v 4.3 software was used for instrument control and data acquisition and Trace Finder v 5.1 software (Thermo Fisher Scientific) was used for data treatment. The identification was made by comparing retention time, the exact masses of the pseudomolecular ion and their fragment ions (maximum deviation of 5 ppm) with a phenolic and phytohormone compound database. Isotopic pattern scores higher than 80% were also required.

The quantification of compounds was performed by the external standard method, using the calibration curve of commercially available standards as previously reported by our group [87,88]. Seven concentrations (10–10,000 ppb) of a standard solution of different compounds were injected: caffeic acid (y = 5.578 × 10^5^x − 2.677 × 10^6^; R^2^ = 0.998), hyperoside (y =1.778 × 10^5^x + 8.738 × 10^5^; R^2^ = 0.997), kaempferol-3,7-di-O-rhamnoside (y = 1.7 × 10^5^x − 5.22 × 10^5^; R^2^ = 0.997), naringenin (y = 1.055 × 10^6^x − 7.949 × 10^6^; R^2^ = 0.996), taxifolin (y = 4.546 × 10^5^x + 4.402 × 10^6^; R^2^ = 0.997), vitexin (y = 3.516 × 10^5^x − 1.28 × 10^6^; R^2^ = 0.999), and isovitexin (y = 3.083 × 10^5^x + 1.134 × 10^8^; R^2^ = 0.997). The results were expressed as µg compound/g dry extract, as mean ± SD. The analysis was done by triplicate. The limit of detection (LOD) and limit of quantification (LOQ) were calculated according to the 3σ/10σ approach, as described by [89], and the linear range were determined (see Table S1 in Supplementary Materials for further details). Only the compounds whose concentrations in TLAE were above the calculated LOQ were considered for quantitative analysis.

The RP-HPLC analysis was performed according to Pinela et al. using a Hitachi LaChrom^®^ (D-7000) Series HPLC system, equipped with an L-7200 automatic injector, a D-7000 interphase, an L-7100 quaternary pump, and a DAD UV–vis L-7455 detector (λ 220–540 nm) [25]. A Symmetry Shield RP 18 column (3.5 μm, 4.6 × 100 mm, Ireland) maintained at 35.0 ± 0.1 °C (L-2350 column oven, Elite LaChrom^®^) was used. The solvents used were: 0.1% *v*/*v* formic acid in water (FA) and acetonitrile (ACN). The elution gradient was 10% ACN (90% FA) to 15% ACN (85% FA) over 5 min, 15–25% ACN (85–75% FA) over 5 min, 25–35% ACN (75–65% FA) over 10 min, 50% ACN (50% FA) for 10 min, and re-equilibration of the column to 10% ACN (90% FA), using a flow rate of 0.5 mL/min. The injection volume was 20 µL, and the run time was 40 min. Data collection and analysis were carried out using HSM D-7000 LaChrom^®^ software version 3.1 (Merck-Hitachi, Darmstadt, Germany). The quantification of punicalagin was performed by the external standard method, using the calibration curve of commercially available punicalagin (Chengdu Biopurify Phytochemicals Ltd., Chengdu, Sichuan, China, 611130). Five concentrations (0.2–0.8 mg/mL) of a punicalagin standard solution in MilliQ water were injected (y = 45302436x − 2674867; R^2^ = 0.999). Then, aliquots of the TLAE dissolved in MilliQ water at different concentrations (1–2.5 mg/mL) were injected. Punicalagin was monitored in the TLAE based on the retention time, UV spectral profile, and characteristic absorbance maxima of the punicalagin standard solution. All samples were filtered through 0.45 µm Branchia^®^ SFNY-245 non-sterile nylon syringe filters. The results were expressed as mg punicalagin/g dry extract, as mean ± SD. The analysis was done by triplicate. The limit of detection (LOD), limit of quantification (LOQ), calculated according to the 3σ/10σ approach as described by [89], and the linear range were determined (see Table S2 in Supplementary Materials for further details).

### 4.5. Evaluation of In Vitro Antioxidant Capacity

#### 4.5.1. DPPH^•^ Free-Radical Scavenging Assay

The 1,1-diphenyl-2-picrylhydrazyl (DPPH^•^) radical scavenging capacity was evaluated using the method proposed by Sánchez-Moreno et al. [90], adapted to a microplate reader [91]. Trolox was used as a positive reference standard. Absorbance was measured at 540 nm, and the results were expressed as inhibitory concentration 50 (IC_50_), or the concentration (µg/mL) capable of scavenging 50% of the DPPH^•^ radical present in the medium. Antioxidant Activity Index (AAI) was calculated according to [27].

#### 4.5.2. ABTS ^•+^ Free-Radical Scavenging Assay

The cation radical 2,2′-azino-bis (3-ethylbenzothiazoline-6-sulfonic acid) diammonium salt (ABTS^•+^) scavenging capacity was evaluated using the method proposed by Re et al. [92], adapted to a microplate reader [93]. Trolox was used as a reference standard. Absorbance was measured at 750 nm, and the results were expressed as IC_50_ (µg/mL), or the concentration capable of scavenging 50% of the ABTS^•+^ present in the medium. Trolox Equivalent Antioxidant Capacity (TEAC) was calculated as follows:

TEAC = IC_50_ Trolox/IC_50_ Sample

#### 4.5.3. H_2_O_2_ Scavenging Assay

The hydrogen peroxide (H_2_O_2_) scavenging capacity was evaluated using the method described by Aruoma et al. [94], with slight modifications for a microplate reader [95]. Trolox was used as a reference standard. Absorbance was measured at 450 nm and results were expressed as IC_50_ (µg/mL), or the concentration capable of scavenging 50% of the H_2_O_2_ present in the medium.

#### 4.5.4. OH^•^ Free-Radical Scavenging Assay

The hydroxyl free radical (OH^•^) scavenging capacity was evaluated using the method described by Guo et al. with slight modifications for a microplate reader [96]. Ascorbic acid was used as a reference standard. Absorbance was measured at 540 nm and results were expressed as IC_50_ (µg/mL), or the concentration capable of scavenging 50% of the OH^•^ present in the medium.

#### 4.5.5. O_2_*^−^*^•^ Free-Radical Scavenging Assay

Superoxide radical (O_2_*^−^*^•^) scavenging capacity was evaluated using the hypoxanthine-xanthine oxidase system according to Aruoma et al. [97], with slight modifications for a microplate reader [98]. Gallic acid was used as a reference standard. Absorbance was measured at 560 nm and results were expressed as IC_50_ (µg/mL), or the concentration capable of scavenging 50% of the O_2_*^−^*^•^ present in the medium.

#### 4.5.6. ^•^NO Free-Radical Scavenging Assay

Nitric oxide (^•^NO) generated from sodium nitroprusside (SNP) was measured using the Griess reagent [99]. Caffeic acid was used as a positive reference control and the absorbance was then measured at 540 nm. The results were compared with the absorbance of sodium nitrite standards treated in the same way with Griess reagent. The results were expressed as IC_50_ (µg/mL), or the concentration capable of scavenging 50% of the ^•^NO present in the medium.

#### 4.5.7. Xanthine Oxidase Inhibition Assay

Xanthine oxidase (XO) enzyme activity was measured by uric acid formation at 295 nm using a UV-visible spectrophotometer at 25 °C according to Nile et al. with slight modifications [100]. Allopurinol was used as a reference standard. Xanthine oxidase (0.8 U/mL in 50 mM phosphate buffer pH 7.4) was used in the experiment. Absorbance was measured at 295 nm for 2 min at 12-s intervals against a blank using a UV-VIS spectrophotometric reader (UV-1800 spectrophotometer, Shimadzu Corporation, Kyoto, Japan). Results were expressed as IC_50_ (µg/mL), or the concentration capable of inhibiting 50% of the enzyme activity.

#### 4.5.8. Fe^2+^ Chelation Assay

Fe^2+^ cation chelation capacity was assessed using the method described by Carter [101], adapted to a microplate reader [102]. EDTA-Na_2_ was used as a positive reference control and the absorbance was measured at 540 nm. The results were expressed as IC_50_ (µg/mL), or the concentration capable of chelating 50% of the Fe^2+^ present in the medium.

### 4.6. Evaluation of Skin-Related Enzyme Inhibitory Activity

#### 4.6.1. Tyrosinase Inhibitory Activity

Tyrosinase inhibitory activity was determined according to No et al. [103]. Kojic acid was used as a reference standard. 10 μL of fungal tyrosinase (initial concentration 1500 units/mL) were used in the experiment. Absorbance was measured at 490 nm using a Multiskan^TM^ FC microplate spectrophotometer (Thermo Fisher Scientific Inc., Waltham, MA, USA). Results were expressed as IC_50_ (µg/mL), or the concentration capable of inhibiting 50% of the enzyme activity.

#### 4.6.2. Elastase Inhibitory Activity

Elastase inhibitory activity was determined by the method described by Tu and Tawata [104] with slight modifications [105]. Quercetin was used as a reference standard. 12.5 μL of porcine pancreatic elastase (initial concentration 0.3 units/mL) were used in the experiment. Absorbance was measured at 410 nm using a Multiskan^TM^ FC microplate spectrophotometer (Thermo Fisher Scientific Inc., Waltham, MA, USA). Results were expressed as IC_50_ (µg/mL), or the concentration capable of inhibiting 50% of the enzyme activity.

#### 4.6.3. Collagenase Inhibitory Activity

Collagenase inhibitory activity was determined following the procedure described by Wang et al. [106] with slightly modifications [105]. Epigallocatechin gallate (EGCG) was used as a reference standard. 100 μL of collagenase from *Clostridium histolyticum* (initial concentration 200 units/mL) were used in the experiment. Absorbance was measured at 550 nm using a Multiskan^TM^ FC microplate spectrophotometer (Thermo Fisher Scientific Inc., Waltham, Massachusetts, USA). Results were expressed as percentage of inhibition at a given concentration (200 µg/mL).

#### 4.6.4. Hyaluronidase Inhibitory Activity

Hyaluronidase inhibitory activity was determined according to the procedure described by Sahasrabudhe and Deodhar [107] with few modifications [105]. Tannic acid was used as a reference standard. A total of 12.5 μL of hyaluronidase from bovine testis (initial concentration 7900 units/mL) were used in the experiment. Absorbance was measured at 585 nm using a Multiskan™ FC microplate spectrophotometer (Thermo Fisher Scientific Inc., Waltham, MA, USA). Results were expressed as IC_50_ (µg/mL), or the concentration required to inhibit 50% of the enzymatic activity.

### 4.7. Cell Culture

Mouse fibroblasts’ (NIH/3T3, ATCC CRL-1658, Manassas, VA, USA) cell line was cultured with Dulbecco’s Modified Eagle’s Medium (DMEM) (12800-017, Gibco (Thermo Fisher Scientific, Waltham, MA, USA)), containing 10% (*v*/*v*) heat-inactivated fetal bovine serum (FBS), 1% (*v*/*v*) antibiotic solution (from a 10.000 U/mL penicillin and 10.000 µg/mL streptomycin stock) (15140-122, Gibco), 3.7 g/L sodium bicarbonate, and 25 mM glucose. The cells were cultured in 75 cm^2^ flasks and maintained in a humidified 5% CO_2_—95% air atmosphere at 37 °C, and the medium was changed every 2–3 days. Cells were detached with TrypLE Express (12605-028, Gibco) when the cells reached 70–80% confluence. Cell morphology was controlled using an inverted light microscope [108].

Human immortalized keratinocytes (HaCaT cell line) were sourced from CLS Cell Lines Service GmbH (Cytion, Eppelheim, Germany). Cells were cultured in 75 cm^2^ flasks and maintained at 37 °C in a humidified atmosphere containing 5% CO_2_ using high-glucose Dulbecco’s Modified Eagle Medium (DMEM) supplemented with 2 mM L-glutamine and enriched with 10% heat-inactivated fetal bovine serum and antibiotics, consisting of 100 U/mL penicillin and 100 µg/mL streptomycin (BIOWEST, Nuaillé, France). The medium was changed every 2–3 days and cells were detached with Trypsin-Ethylenediamine tetraacetic acid (EDTA) solution 5X (BIOWEST, Nuaillé, France) when the cells reached 70–80% confluence. Cell morphology was controlled using an Olympus CKX41 inverted light microscope.

### 4.8. Cell Viability

The effect of different concentrations of the TLAE on the viability of fibroblasts and HaCaT cells was carried out using the resazurin (Sigma-Aldrich, St. Louis, MO, USA) reduction assay according to Alves-Silva et al. [108], [109] and the 3-(4,5-dimethyl-2-thiazolyl)-2,5-diphenyl tetrazolium bromide (MTT) (Calbiochem, Darmstadt, Hesse, Germany) assay according to Avila-Roman et al. [109], [108], respectively. Briefly, fibroblasts (5 × 10^4^ cells/mL) were seeded in 48-well plates. After an overnight stabilization, 25–600 μg/mL of the TLAE diluted in culture medium from a stock solution made in distilled H_2_O/DMSO (9.5:0.5) was added. After 24 h, the medium was removed and fresh medium containing resazurin (500 µM; 1:10) was added for 2 h. The absorbance at 570 nm with a reference filter 620 nm was registered in an automated plate reader (SLT, Salzburg, Austria). Cell viability was determined using the following equation:

Cell viability (%) = (Abs_Exp_/Abs_CT_) × 100

where Abs_Exp_ is the absorbance (difference between 570 and 620 nm) in the different experimental conditions and Abs_CT_ is the absorbance in control cells (no TLAE).

Regarding the MTT assay, 100 μL/well of HaCaT cells were seeded at 10^4^ cells/well in 96-well plates and incubated for 24 h. Subsequently, the medium was replaced with TLAE-containing medium (12.5–100 µg/mL), and cells were incubated for 24 h. After exposure and medium removal, 100 µL of MTT solution (0.25 mg/mL) was added, and plates were incubated for 4 h at 37 °C. Then the reagent was removed, and the formazan crystals were dissolved with DMSO (200 μL) and 0.1 M glycine buffer pH 10.5 (25 μL). The absorbance was measured at 550 nm using a microplate spectrophotometer iMark™ Microplate Reader (BIO-RAD, Hercules, CA, USA). Cell viability was determined using the same equation as in resazurin assay.

### 4.9. Cell Migration

The scratch wound assay, adapted from the method of Martinotti and Ranzato with minor modifications, was performed [110]. Briefly, NIH/3T3 fibroblasts were plated at a density of 3 × 10^5^ cells/mL in 12-well plates and cultured for 24 h. A vertical artificial wound (scratch) was made in the cell monolayer using a 20 µL pipette tip, and detached cells were removed by washing with sterile PBS. The remaining cells were cultured in medium containing 2% FBS, with or without TLAE at different concentrations (100–25 μg/mL). Images of the wound area were captured by phase-contrast microscopy (Carl Zeiss, Oberkochen, Germany) at a magnification of 10× and 5× immediately after scratching (0 h) and again after 18 h of incubation. The wound area was quantified using an ImageJ2 version 2.16.0/1.54g/Fiji plugin as described by Suarez-Arnedo et al. [111]. The percentage of the wound closure was calculated by the following formula:

Closed wound area (%) = 100 − [(OWA_18h_/OWA_0h_) × 100]

where OWA_18h_ is the open wound area after 18 h of incubation and OWA_0h_ is the open wound area immediately after scratching.

### 4.10. Antifungal Activity

The antifungal activity of the extract was evaluated against several pathogenic strains: three dermatophyte clinical strains isolated from nails and skin (*Epidermophyton floccosum* FF9, *Microsporum canis* FF1, and *Trichophyton mentagrophytes* FF7), four dermatophyte reference strains (*M. gypseum* CECT 2908, Valencia, Spain; *T. mentagrophytes* var. *interdigitale* CECT 2958, Valencia, Spain; *T. rubrum* CECT 2794, Valencia, Spain and *T. verrucosum* CECT 2992, Valencia, Spain), two clinical *Candida* strains isolated from recurrent cases of vulvovaginal and oral candidiasis (*C. krusei* H9 and *C. guilliermondii* MAT23), three *Candida* reference strains (*C. albicans* ATCC 10231, Manassas, VA, USA, *C. parapsilopsis* ATCC 90018, Manassas, VA, USA, and *C. tropicalis* ATCC 13803 Manassas, VA, USA). All of the strains were subcultured in Sabouraud dextrose agar (SDA) or potato dextrose agar (PDA) (Oxoid—Thermo Fisher Scientific, Waltham, MA, USA) before each test, in order to ensure optimal growth conditions and purity.

A macrodilution method was used to evaluate the minimum inhibitory concentrations (MICs) and the minimum lethal concentrations (MLCs) of the TLAE, according to the Clinical and Laboratory Standards Institute (CLSI) reference protocols M27-A3 and M38-A2 for yeasts and filamentous fungi, respectively [112,113,114]. Fluconazole was used as a reference standard.

### 4.11. Antibiofilm Activity Against Epidermophyton floccosum FF9 Biofilms

#### 4.11.1. Effect on Biofilm Formation

The effect of TLAE on *E. floccosum* FF9 biofilm formation was studied according to Ali et al. [115] with slight modifications previously detailed by our research group [116].

#### 4.11.2. Effect Towards Mature Biofilms

The potential of the extract to disrupt mature biofilms was determined according to Ali et al. [115] with slight modifications previously detailed by our research group [116].

#### 4.11.3. Biofilm Mass Quantification

Biofilm biomass was measured by crystal violet staining. Dermatophyte biofilms were stained according to the method described by Castelo-Branco et al. [117]. Absorbance was measured at 620 nm using a Multiskan™ FC microplate spectrophotometer (Thermo Fisher Scientific Inc., Waltham, Massachusetts, USA).

#### 4.11.4. Biofilm Extracellular Matrix Quantification

The extracellular matrix (ECM) of dermatophyte biofilms was quantified using safranin red according to the method described by Costa-Orlandi et al. [118]. Absorbance was measured at 520 nm using a Multiskan™ FC microplate spectrophotometer (Thermo Fisher Scientific Inc., Waltham, MA, USA).

#### 4.11.5. Biofilm Metabolic Activity Evaluation

The metabolic activity of the biofilms was assessed using the XTT reduction assay (2,3-Bis(2-methoxy-4-nitro-5-sulfophenyl)-2H-tetrazolium-5-carboxanilide) according to Alves et al. [119]. Absorbance was measured at 490 nm using a Multiskan™ FC microplate spectrophotometer (Thermo Fisher Scientific Inc., Waltham, MA, USA).

### 4.12. Statistical Analysis

Data were processed using GraphPad Prism version 9.5.1 (GraphPad Software, San Diego, CA, USA) and expressed as mean ± SD. Normality in the distribution of the data was assessed using the Shapiro–Wilk, Kolmogorov–Smirnov, and D’Agostino–Pearson tests. To analyze the data, one-way or two-way ANOVA followed by Dunnett’s test was used for comparisons involving three or more groups with normal distribution. For comparisons between two groups, either the Student’s *t*-test or Welch’s *t*-test was applied. Non-parametric tests were used when the data did not meet the assumptions of normality. Statistical significance was set at *p* < 0.05 (*), *p* < 0.01 (**), and *p* < 0.001 (***) for all analyses.

## 5. Conclusions

Overall, *Tuberaria lignosa* (Sweet) Samp. leaf aqueous extract, which is rich in phenolic compounds, particularly punicalagin, has demonstrated a potent in vitro antioxidant activity against a broad range of oxidative agents, including reactive oxygen and nitrogen species, transition metals, synthetic radicals, and pro-oxidant enzymes, likely attributable to its phenolic composition. The extract was non-cytotoxic to HaCaT cells and NIH/3T3 fibroblasts at concentrations up to 100 µg/mL and 200 µg/mL, respectively. At 25 µg/mL, it promoted wound closure comparable to the control, while from 50 µg/mL to 100 µg/mL, it inhibited fibroblast migration in a dose-dependent manner. The extract also effectively inhibited enzymes involved in extracellular matrix degradation and in processes related to skin aging. It exhibited fungistatic and fungicidal activity against several dermatophyte species and antibiofilm effects against *Epidermophyton floccosum,* supporting its potential relevance in dermatological applications. This study opens the way for further phytochemical, preclinical, and clinical research aimed at treating various skin conditions, and brings new insight to the broad scientific potential of this understudied species.

## Figures and Tables

**Table 1:** Total phenolic, flavonoid, hydroxycinnamic acid, proanthocyanidin, and tannin content in the *Tuberaria lignosa* (Sweet) Samp. aqueous leaf extract.

Assay	Content
Total Phenolic Content (mg GAE/g dry extract)	425.33 ± 9.02
Total Flavonoid Content (mg RE/g dry extract)	181.21 ± 6.04
Total Hydroxycinnamic Acid Content (mg CAE/g dry extract)	28.88 ± 3.08
Total Proanthocyanidin Content (mg CE/g dry extract)	38.64 ± 4.01
Total Tannin Content (TRP % *w/w*)	71.96 ± 2.25

GAE—Gallic acid equivalents; RE—Rutin equivalents; CAE—Caffeic acid equivalents; CE—Catechin equivalents; TRP—Tannin-related phenolics. Data are expressed as mean ± standard deviation (*n* = 3 performed in duplicate).

**Table 2:** Phenolic compounds in *T. lignosa* aqueous leaf extract analyzed by UHPLC-HRMS/MS.

RT (min)	Molecular Formula	Expected *m*/*z*	Measured *m*/*z*	Error (ppm)	MS/MS Fragments	Attribution	Content µg/g Dry Extract ^1^
5.52	C_21_H_20_O_10_	431.09837	431.098465	0.220285	77.03976; 117.0347; 283.06152; 311.05661; 341.06689	Isovitexin	375.44 ± 7.49
5.32	C_21_H_20_O_10_	431.09837	431.09831	0.220285	77.03976; 117.0347; 283.06152; 311.05661; 341.06689	Vitexin	216.12 ± 2.54
5.66	C_21_H_20_O_12_	463.0882	463.08832	0.25453	227.03548; 243.02995; 255.03014; 271.02521; 300.0278	Hyperoside	37.73 ± 1.67
5.99	C_27_H_30_O_14_	577.15628	577.15613	−0.26348	117.03459; 183.04515; 255.0299; 285.04046; 430.09054	Kaempferol-3,7-O-α-di- rhamnopyranoside	27.30 ± 1.65
6.82	C_15_H_12_O_5_	271.0612	271.061235	0.123665	65.00333; 83.01394; 107.01404; 119.0503; 151.00375	Naringenin	N/A
4.03	C_9_H_8_O_4_	179.03498	179.03497	−0.0595975	107.05025; 134.03757; 135.04532; 179.03532	Caffeic acid	N/A
5.02	C_15_H_12_O_7_	303.05103	303.050975	−0.16626	57.03462; 125.02451; 151.00342; 175.04033; 285.04071	Taxifolin	N/A
0.52	C_7_H_12_O_6_	191.05611	191.056085	−0.137765	85.02961; 93.03466; 109.02957; 127.04015; 173.0459	Quinic acid	N/A
1.11	C_7_H_6_O_5_	169.01425	169.0141875	−0.3735825	69.03467; 79.01904; 81.03464; 97.02959; 125.02452	Gallic acid	N/A
1.83	C_15_H_14_O_7_	305.06668	305.06662	−0.1970925	109.02959; 125.02451; 137.02457; 167.03516; 219.06656	(-) Gallocatechin	N/A
2.20	C_7_H_6_O_4_	153.01933	153.01931	−0.13072	65.00337; 81.03465; 91.01901; 108.02183; 109.0296	Protocatechuic acid	N/A
3.13	C_48_H_28_O_30_	1083.05926	1083.06042	1.07104	300.99921; 600.99017; 781.05371	Punicalagin	N/A
3.31	C_30_H_26_O_12_	577.13515	577.13495	−0.34874	109.02943; 125.02448; 161.02473; 289.07266; 407.07812	Procyanidin B1	N/A
3.59	C_15_H_14_O_6_	289.07176	289.0718625	0.3503175	109.02957; 123.04529; 137.02441; 203.0719; 245.0822	Catechin	N/A
4.50	C_8_H_8_O_3_	151.04007	151.040025	−0.280595	92.02686; 108.02182; 136.01671; 151.0403	Vanillin	N/A
4.82	C_9_H_8_O_3_	163.04007	163.040055	−0.0727675	65.03967; 91.05544; 93.03464; 104.02702; 119.05034	p-Coumaric acid	N/A
5.78	C_7_H_6_O_3_	137.02442	137.02435	−0.516605	65.03975; 93.03467; 137.02455	Salicylic acid	N/A
5.61	C_15_H_12_O_6_	287.05611	287.056165	0.200665	65.00336; 83.01392; 125.02451; 177.05606; 259.06152	Aromadendrin	N/A
5.67	C_14_H_6_O_8_	300.99899	300.99887	−0.39585	117.03472; 145.02966; 173.0246; 201.01962; 283.99649	Ellagic acid	N/A
6.32	C_15_H_10_O_7_	301.03538	301.0352275	−0.5151725	65.00338; 83.01395; 149.02454; 151.00392	Morin	N/A
6.57	C_15_H_20_O_4_	263.12888	263.1288325	−0.19019	122.03747; 153.09236; 203.10808; 204.11569; 219.13937	Abscisic acid	N/A
7.23	C_15_H_10_O_6_	285.04046	285.04047	0.0221325	65.00337; 93.03457; 117.03468; 159.04544; 187.04065	Kaempferol	N/A

^1^ Quantification data are expressed as mean ± standard deviation (*n* = 3). N/A: not quantified.

**Table 3:** In vitro antioxidant activity (IC_50_ µg/mL) of *T. lignosa* aqueous leaf extract.

Sample	Free Radicals		Reactive Oxygen and Nitrogen Species (ROS; RNS)				Enzymes	Transition Metals
	^△^DPPH^•^	^▽^ABTS ^•+^	H_2_O_2_	OH^•^	O_2_^−•^	^•^NO	Xanthine Oxidase	Fe^2+^
TLAE	9.65 ± 0.49 ^c^	4.97 ± 0.47 ^ns^	9.43 ± 0.44 ^ns^	313.51 ± 24.82 ^b^	8.81 ± 0.71 ^c^	35.22 ± 1.33 ^c^	27.6 ± 1.59 ^c^	10.54 ± 0.35 ^c^
Allopurinol	-	-	-	-	-	-	0.33 ± 0.01	-
Ascorbic acid	-	-	-	250.36 ± 0.920	-	-	-	-
EDTA-Na_2_	-	-	-	-	-	-	-	7.83 ± 0.30
Caffeic acid	-	-	-	-	-	7.02 ± 0.22	-	-
Trolox	6.25 ± 0.07	5.37 ± 0.15	9.93 ± 0.45	-	-	-	-	-
Gallic acid	-	-	-	-	4.70 ± 0.73	-	-	-

Data are expressed as mean ± standard deviation (*n* = 3 performed in duplicate). Statistical analysis was performed either by Student’s *t*-test for independent samples with equal variances or Welch’s *t*-test for samples with unequal variances: ^ns^ *p* ≥ 0.05, ^b^ *p* < 0.01, ^c^ *p* < 0.001. ^△^AAI_TLAE_: 4.56 ± 0.21; ^△^AAI_Trolox_: 7.06 ± 0.07; ^▽^TEAC_TlAE_: 1.08 ± 0.096.

**Table 4:** In vitro skin-related enzyme inhibitory activity of *T. lignosa* aqueous leaf extract.

Sample	Skin-Related Enzymes			
	% Inhibition at 200 µg/mL	IC_50_ (µg/mL)		
	Collagenase	Elastase	Hyaluronidase	Tyrosinase
TLAE	58.49 ± 4.08 ^ns^	219.02 ± 11.31 ^b^	>675	67.48 ± 2.78 ^c^
EGCG	62.52 ± 1.86	-	-	-
Kojic acid	-	-	-	22.38 ± 1.01
Quercetin	-	100.26 ± 8.66	-	-
Tannic acid	-	-	171.96 ± 11.28	-

Data are expressed as mean ± standard deviation (*n* = 3 performed in duplicate). Percentage of inhibition at a specific concentration (200 μg/mL) is presented for the collagenase inhibition assay in comparison with EGCG (Epigallocatechin gallate), while IC50 values (μg/mL) are reported for elastase, hyaluronidase, and tyrosinase inhibition assays. Statistical analysis was performed either by Student’s *t*-test for independent samples with equal variances or Welch’s *t*-test for samples with unequal variances: ^ns^ *p* ≥ 0.05, ^b^ *p* < 0.01, and ^c^ *p* < 0.001.

**Table 5:** Fungistatic and fungicidal activity of *T. lignosa* aqueous leaf extract (TLAE) against different dermatophytes and yeast strains.

Strains	TLAE		Fluconazole	
	MIC	MLC	MIC	MLC
*Epidermophyton floccosum* FF9	100	100	16	16
*Microsporum canis* FF1	100	100	128	128
*Microsporum gypseum* CECT 2908	400	400	128	>128
*Trichophyton mentagrophytes* FF7	50	50	32	32–64
*Trichophyton mentagrophytes var interdigitale* CECT 2958	100	100–200	128	>128
*Trichophyton rubrum* CECT 2794	100–200	200	16	64
*Trichophyton verrucosum* CECT 2992	>1000	-	>128	-
*Candida krusei* H9	1000	>1000	64	64–128
*Candida albicans* ATCC 10231	>1000	-	1	>128
*Candida guilliermondii* MAT23	>1000	-	4	>128
*Candida parapsilosis* ATCC 90018	>1000	-	1	2
*Candida tropicalis* ATCC 13803	>1000	-	4	>128

Data are expressed as μg/mL (*n* = 3 performed in duplicate). MIC: Minimum inhibitory concentration; MLC: Minimum lethal concentration. Ranges of values indicate the variation observed in repeated assays.

## Data Availability

The data presented in this study are available on request from the corresponding author.

## Supplementary Materials

The following supporting information can be downloaded at: https://www.mdpi.com/article/10.3390/plants14152299/s1.

## Abbreviations

The following abbreviations are used in this manuscript:

TLAE	*Tuberaria lignosa* (Sweet) Samp. leaf aqueous extract
ECM	Extracellular matrix
GAE	Gallic acid equivalents
RE	Rutin equivalents
CAE	Caffeic acid equivalents
CE	Catechin equivalents
TRP	Tannin-related phenolics
UHPLC-HRMS/MS	Ultra-high-performance liquid chromatography coupled with high-resolution mass spectrometry
HPLC-DAD	High-performance liquid chromatography with diode array detection
IC50	Inhibitory concentration 50
DPPH	1,1-diphenyl-2-picrylhydrazyl
ABTS	2,2′-azino-bis (3-ethylbenzothiazoline-6-sulfonic acid) diammonium salt
EDTA	Ethylenediaminetetraacetic acid
XO	Xanthine oxidase
MTT	3-(4,5-dimethyl-2-thiazolyl)-2,5-diphenyl tetrazolium bromide
XTT	2,3-Bis(2-methoxy-4-nitro-5-sulfophenyl)-2H-tetrazolium-5-carboxanilide
MIC	Minimum inhibitory concentration
MLC	Minimum lethal concentration
