# Supplementary material for: Phytochemical Analysis and Therapeutic Potential of Tuberaria lignosa (Sweet) Samp. Aqueous Extract in Skin Injuries
Source: Plants (Basel). 2025 Jul 25;14(15):2299. doi: 10.3390/plants14152299 (PMC12348581; doi:10.3390/plants14152299)
Supplement: Supplementary file 1 [file plants-14-02299-s001.zip › Supplementary Document S1.pdf]

Supplementary material

Document S1: Detailed information on the methodology used in the article "Phytochemical analysis and therapeutic potential of *Tuberaria lignosa* (Sweet) Samp. aqueous extract in skin injuries".

1. Materials and Methods Detailed information

1.1. Detailed information about the in vitro antioxidant capacity evaluation

1.1.1. H_2O_2 scavenging assay

The hydrogen peroxide (H_2O_2) scavenging capacity was evaluated as described in the original article linked to this supplementary material. Briefly, 235 µL of sodium phosphate buffer (0.01 M pH 7.4), 3 µL of the sample or standard and 26 µL of H_2O_2 (1 mM) were mixed in a 96 well plate. After 30 minutes at ambient temperature, 13 µL of guaiacol (0.2% v/v in distilled water) and 3 µL of horseradish peroxidase (900 U/mL in phosphate buffer) were added to the mixture. After 10 min the absorbance was measured at 450 nm. Trolox was used as a reference standard. H_2O_2 scavenging capacity was calculated as:

$$H_2O_2 \text{ scavenging capacity (\%)} = [1 - (A_1 - A_2) / A_0] \times 100$$

Where A_0 is the absorbance of the control, A_1 is the absorbance of the sample or standard, and A_2 is the absorbance of the sample or standard blank. The results were expressed as IC_{50} (µg/mL), or the concentration capable of scavenging 50% of the H_2O_2 present in the medium.

1.1.2. $OH^\bullet$ free-radical scavenging assay

The hydroxyl free radical ($OH^\bullet$) scavenging capacity was evaluated as described in the original article linked to this supplementary material. Briefly, 30 µL of sodium salicylate (20 mM), 100 µL of ferrous sulfate (1.5 mM), 50 µL of the reference sample or positive control at different concentrations, and 70 µL of H_2O_2 (6 mM) were mixed in a microtiter plate in the following order. After the addition of all reagents, the plate was kept at 37 ºC for 1 hour and the absorbance was measured at 540 nm. Ascorbic acid was used as a reference standard. The $OH^\bullet$ scavenging capacity was calculated as:

$$OH^\bullet \text{ scavenging capacity (\%)} = [1 - (A_1 - A_2) / A_0] \times 100$$

Where A0 is the absorbance of the negative control, A1 is the absorbance of the sample or standard with salicylate, and A2 is the absorbance of the blank of the sample or standard. The results were expressed as IC_{50} (µg/mL), or the concentration capable of scavenging 50% of the $OH^\bullet$ present in the medium.

1.1.3. $O_2^{-\bullet}$ free-radical scavenging assay

Superoxide radical ($O_2^{-\bullet}$) scavenging capacity was evaluated as described in the original article linked to this supplementary material. Briefly, in a 96-well plate, 62 µL of phosphate buffer (50 mM pH 7.4), 10 µL of ethylenediaminetetraacetic acid disodium (EDTA-Na_2) (15 mM), 15 µL of hypoxanthine (3 mM), 25 µL of nitroblue tetrazoyl (NBT) (0.6 mM), 12 µL of the reference sample or standard at different concentrations, and 25 µL of xanthine oxidase (0.36 U/mL) were added. After addition of all reagents, absorbance was measured at 560 nm at 5-minute intervals for 40 minutes. Gallic acid was used as a positive reference control. $O_2^{-\bullet}$ scavenging capacity was calculated as:

$$O_2^{-\bullet} \text{ scavenging capacity (\%)} = [1 - (M_a / M_c)] \times 100$$

Where Mc is the slope (increase in absorbance versus time) of the negative control and Ma is the slope of the reference sample or standard. Results were expressed as IC_{50} (μg/mL), or the concentration capable of scavenging 50% of the $O_2^{-\bullet}$ present in the medium.

1.1.4. Xanthine oxidase inhibition assay

Xanthine oxidase (XO) enzyme activity was measured as described in the original article linked to this supplementary material. Briefly, 100 μL of xanthine oxidase (0.8 U/mL in 50 mM phosphate buffer pH 7.4) and 10 μL of the sample or reference standard were mixed in a spectrophotometric cuvette in the following order. After 15 minutes at room temperature, 780 μL phosphate buffer (50 mM, pH 7.4), 100 μL EDTA (10 mM) and 10 μL xanthine (10 mM) were added. Immediately thereafter, the absorbance of the preparation was measured at 295 nm for 2 minutes at 12-second intervals against a blank using a UV-VIS spectrophotometric reader (UV-1800 spectrophotometer, Shimadzu Corporation, Kyoto, Japan). Allopurinol was used as a positive reference control. Both the sample and allopurinol were dissolved in a mixture of phosphate buffer and DMSO (final concentration of DMSO in the reaction mixture < 1% v/v). A blank for the sample or standard (without enzyme) and a negative control were included. The inhibitory capacity of xanthine oxidase was calculated as follows:

$$\text{Inhibition (\%)} = [1 - (M_a / M_c)] \times 100$$

Where Mc is the slope (increase in absorbance versus time) of the negative control and Ma is the slope of the reference sample or standard. Results were expressed as IC_{50} (μg/mL), or the concentration capable of inhibiting 50% of the enzyme activity.

1.2. Detailed information about the skin-related enzyme inhibitory activity evaluation

1.2.1. Tyrosinase inhibitory activity

Tyrosinase inhibitory activity was determined as described in the original article linked to this supplementary material. In a 96-well plate, the following reagents were added in the following order: 110 μL 0.1 M PBS (pH 6.8), 10 μL sample or standard in PBS (final concentrations 500-6.25 μg/mL), 10 μL fungal tyrosinase (Sigma-Aldrich Chemical Co., St. Louis, MO, USA) in PBS (initial concentration 1500 units/mL) and 20 μL 1.5 mM L-tyrosine in PBS. After the addition of the last reagent, the mixture was incubated at 37°C for 15 minutes, after which the reaction was stopped by incubating the plate on ice for 1 minute. Absorbance was measured at 490 nm using a Multiskan™ FC microplate spectrophotometer (Thermo Fisher Scientific Inc., Waltham, Massachusetts, USA). Kojic acid (final concentrations 100-6.25 μg/mL) was used as a positive reference control. A sample or standard blank (without enzyme), a negative control (without sample or standard) and a negative control blank (without enzyme and without sample or standard) were included. The sample stock solution was prepared in distilled H_2O/DMSO (9.5:0.5) and the standard in PBS. In no case did the final concentration of DMSO in the reaction mixture exceed 0.5%. The percentage of tyrosinase inhibition was calculated as follows:

$$\text{Inhibition (\%)} = [1 - ((A1a – A1b) / (A0a - A0b))] \times 100$$

Where A1a is the absorbance of the reference sample or standard, A1b is the absorbance of the sample blank or standard (without enzyme), A0a is the absorbance of the negative control

and A0b is the absorbance of the negative control blank. The results were expressed as IC_{50} (μg/ml), or the concentration capable of inhibiting 50% of the enzyme activity.

1.2.2. Elastase inhibitory activity

Elastase inhibitory activity was determined as described in the original article linked to this supplementary material. In a 96-well plate, the following reagents were added in the following order 50 μl of 0.2 M Tris-HCl buffer (pH 8), 25 μl of sample or standard in Tris-HCl buffer (final concentrations 1000-62.5 μg/mL in the final reaction mixture), 12.5 μl of 10 mM N-succinyl para-nitroanilide (AAAPVN) in Tris-HCl buffer. This reaction mixture was incubated for 15 min at 25 °C and, after the addition of 12.5 μl of porcine pancreatic elastase (Sigma-Aldrich Chemical Co., St. Louis, MO, USA) in Tris-HCl buffer (initial concentration 0.3 units/ml), the absorbance was measured at 410 nm using a Multiskan™ FC microplate spectrophotometer (Thermo Fisher Scientific Inc., Waltham, Massachusetts, USA). The final reaction mixture was then re-incubated for a further 15 min at 25 °C. After this time, the absorbance was measured again at 410 nm. Quercetin (final concentrations 200-6.25 μg/mL) in Tris-HCl buffer was used as a positive reference control. A negative control (no sample or standard) was included. The sample stock solution was prepared in distilled H_2O/DMSO (9.5:0.5) and the standard in DMSO. In no case did the final concentration of DMSO in the final reaction mixture exceed 0.5%. The percentage inhibition of elastase was calculated as follows:

$$\text{Inhibition (\%)} = [1 - ((A1a – A1b) / (A0a - A0b))] \times 100$$

Where A1a is the absorbance of the reference sample or standard after the second incubation, A1b is the absorbance of the sample or standard after the first incubation, A0a is the absorbance of the negative control after the second incubation and A0b is the absorbance of the negative control after the first incubation. The results were expressed as IC_{50} (μg/ml), or the concentration capable of inhibiting 50% of the enzyme activity.

1.2.3. Collagenase inhibitory activity

Collagenase inhibitory activity was determined as described in the original article linked to this supplementary material. In eppendorfs of 1 ml capacity, these reagents were added in the following order: 1 mg of collagen impregnated with azo dye (Azocoll™, Sigma-Aldrich Chemical Co, St. Louis, MO, USA), 800 μl of 0.1 M Tris-HCl buffer (pH 7), 100 μl of sample or standard in Tris-HCl buffer and 100 μl of collagenase from *Clostridium histolyticum* (Sigma-Aldrich Chemical Co., St. Louis, MO, USA) in Tris-HCl buffer (initial concentration 200 units/ml). After this, tubes were incubated in a water bath at 43 °C for 1 hour. Subsequently, the tubes were homogenized and centrifuged at 3000 rpm for 10 minutes. Finally, the supernatant was transferred to a microtiter plate and absorbance was measured at 550 nm using a Multiskan™ FC microplate spectrophotometer (Thermo Fisher Scientific Inc., Waltham, Massachusetts, USA). Epigallocatechin gallate (EGCG) in Tris- HCl buffer was used as a positive reference control. A blank for the sample or standard (without enzyme), a negative control (without sample or standard) and a blank for the negative control (without enzyme and without sample or standard) were included. The sample stock solution was prepared in distilled H_2O/DMSO (9.5:0.5) and the standard in Tris-HCl. In no case did the final concentration of DMSO in the reaction mixture exceed 0.5%. The percentage of inhibition of collagenase was calculated as:

$$\text{Inhibition (\%)} = [1 - ((A1a – A1b) / (A0a - A0b))] \times 100$$

Where A1a is the absorbance of the reference sample or standard, A1b is the absorbance of the sample blank or standard (without enzyme), A0a is the absorbance of the negative control and A0b is the absorbance of the negative control blank. The results were expressed as percentage of inhibition at a given concentration (200 μg/mL).

1.2.4. Hyaluronidase inhibitory activity

Hyaluronidase inhibitory activity was determined as described in the original article linked to this supplementary material. The following reagents were added in 1 ml eppendorfs in the following order 12.5 μL sample or standard in 0.1 M acetate buffer pH 3.6 (final concentrations 675-32.5 μg/mL in the 25 μL reaction mixture) and 12.5 μL hyaluronidase (initial concentration 7900 units/ml) from bovine testis (Sigma-Aldrich Chemical Co., St. Louis, MO, USA) in acetate buffer. This mixture was incubated in a water bath at 37ºC for 20 minutes. After this time, 25 μL of an aqueous 12.5 mM $CaCl_2$ solution was added and the mixture was incubated for a further 20 minutes at 37 ºC in a water bath. After this time, 62.5 μL of a solution of hyaluronic acid (Sigma-Aldrich Chemical Co., St. Louis, MO, USA) 1.2 mg/ml in acetate buffer was added and the mixture was incubated again for 40 minutes at 37 ºC. Then 25 μl of an aqueous solution of 0.2 M potassium tetraborate tetrahydrate and 25 μl of 0.4 M NaOH were added. At this stage, the reaction mixture was maintained for 3 minutes at 100 °C in a water bath and then cooled rapidly in an ice bath. Once the reaction mixture reached a lower temperature, 750 μL of a 0.01 M solution of 4-(dimethylamino)benzaldehyde (DMAB) in glacial acetic acid (CH_3COOH) 17.47 M was added (prepared from a 0.08 M DMAB stock solution in CH_3COOH 17.47 M/HCl 10 M at a ratio of 8.88:1.12). Finally, after homogenizing the contents of the Eppendorf tubes and incubating the reaction mixture for an additional 20 minutes at 37 °C, 200 μL of the solution was transferred to a microtiter plate, and absorbance was measured at 585 nm using a Multiskan™ FC microplate spectrophotometer (Thermo Fisher Scientific Inc., Waltham, Massachusetts, USA). Tannic acid in acetate buffer (final concentrations ranging from 900–12.5 μg/mL) was used as a reference positive control. A sample or standard blank (without enzyme), a negative control (without sample or standard), and a blank for the negative control (without enzyme, sample, or standard) were included. The stock solution of the sample was prepared in H_2O/DMSO at a 9.5:0.5 ratio, while the standard was prepared in acetate buffer. In all cases, the final concentration of DMSO in the reaction mixture did not exceed 0.5%. The percentage of hyaluronidase inhibition was calculated as follows:

$$\text{Inhibition (\%)} = [1 - ((A1a – A1b) / (A0a - A0b))] \times 100$$

Where A1a is the absorbance of the sample or reference standard, A1b is the absorbance of the sample or standard blank (without enzyme), A0a is the absorbance of the negative control, and A0b is the absorbance of the negative control blank. Results were expressed as IC_{50} (μg/ml), or the concentration required to inhibit 50% of the enzymatic activity.

1.3. Detailed information about the antifungal activity evaluation

The antifungal activity of the extract was evaluated against several pathogenic strains: three dermatophyte clinical strains isolated from nails and skin (*Epidermophyton floccosum* FF9, *Microsporum canis* FF1, and *Trichophyton mentagrophytes* FF7), four dermatophyte reference strains (*M. gypseum* CECT 2908, Valencia, Spain; *T. mentagrophytes* var. *interdigitale* CECT 2958, Valencia, Spain; *T. rubrum* CECT 2794, Valencia, Spain and *T. verrucosum* CECT 2992, Valencia, Spain), two clinical *Candida* strains isolated from recurrent cases of vulvovaginal and oral candidiasis (*C. krusei* H9 and *C. guilliermondii* MAT23), three *Candida* reference strains (*C. albicans* ATCC 10231, Manassas, VA, USA, *C. parapsilopsis* ATCC 90018, Manassas, VA, USA, and *C. tropicalis* ATCC

13803 Manassas, VA, USA). All of the strains were subcultured in Sabouraud dextrose agar (SDA) or Potato dextrose agar (PDA) (Oxoid—Thermo Fisher Scientific, Waltham, MA, USA) before each test, in order to ensure optimal growth conditions and purity.

A macrodilution method was used to evaluate the Minimum Inhibitory Concentrations (MICs) and the Minimum Lethal Concentrations (MLCs) of the TLAE, according to the Clinical and Laboratory Standards Institute (CLSI) reference protocols M27-A3 and M38-A2 for yeasts and filamentous fungi, respectively. Briefly, 100 μL of serial twofold dilutions of the extract from a 10 mg/ml stock solution in distilled H_2O/DMSO (9:1) were distributed into glass test tubes (to reach final concentrations from 1000 μg/mL to 25 μg/mL). Fungal inoculum suspensions were prepared from SDA or PDA cultures at 0.5 McFarland units in sterile NaCl 0.9% (w/v) and then diluted in RPMI-1640 without glutamine and with 165 mM 3-(N-morpholino)propanesulfonic acid (MOPS) (Sigma-Aldrich, St. Louis, MO, USA) pH 7.0 to a concentration of $1–2 \times 10^4$ CFU/mL, which was then added to the test tubes containing the TLAE. The test tubes were incubated at 35 °C for 48 hours/72 hours for *Candida* spp., or at 30 °C for 7 days for dermatophytes. Afterward, tubes were assessed for fungal growth, and the lowest concentration without visible growth (MIC) was determined. The lowest concentration where no growth was observed after plating the negative tubes in SDA for 7 days at 30 ºC or 48 hours/72 hours at 35 ºC was considered the MLC in the case of dermatophytes or *Candida* spp., respectively. The final DMSO concentrations never exceeded 1% v/v. Extract-free positive controls (inoculum and DMSO 1% v/v), and negative controls (RPMI medium without inoculum) were also included. Fluconazole (0.25 to 128 μg/mL) was used as a reference standard. This was performed as described in the original article linked to this supplementary material.

1.4. Detailed information about the antibiofilm activity evaluation

1.4.1. Effect on biofilm formation

The effect of TLAE on *E. floccosum* FF9 biofilm formation was studied as described in the original article linked to this supplementary material. Briefly, *E. floccosum* FF9 inoculums were prepared from 7-day-old cultures in SDA by adding sterile 0.9% (w/v) NaCl and vigorously vortexed to detach conidia. The saline suspension was then transferred to a new sterile tube and left to settle for 5 minutes to allow the separation of the conidia from hyphae. The supernatant was then collected to another sterile tube and turbidity was adjusted to 1 unit of McFarland scale, containing approximately 1×10^6 conidia/mL. Afterward, 200 μL of conidia suspension was added to sterile 96-well flat-bottom polystyrene microtiter plates and incubated at 37 °C for 3 hours to allow for conidia to adhere. Then, saline was removed, and wells were washed with sterile PBS (pH 7.4) to remove non-adherent cells. Subsequently, 200 μL of RPMI-1640 with MOPS containing different concentrations of the aqueous extract (final concentrations ranging from 100 μg/mL to 12.5 μg/mL) was added and incubated for 72 hours at 37 °C. Negative and positive controls containing non-inoculated medium and extract-free inoculated medium (DMSO 1% v/v) were included, respectively. The final DMSO concentration never exceeded 1% v/v.

1.4.2. Effect towards mature biofilms

The potential of the extract to disrupt mature biofilms was determined as described in the original article linked to this supplementary material. Briefly, 200 μL of *E. floccosum* FF9 conidia suspensions (prepared as previously described) were left to adhere for 3 hours at 37 °C in sterile 96-well flat-bottom polystyrene microtiter plates. After saline removal, cells were washed with PBS to remove non-adherent cells, and 200 μL of sterile RPMI medium was added to the plates and left to incubate for 72 hours at 37 °C. After removing the medium and washing with sterile PBS (pH 7.4), 200 μL of RPMI-1640 with MOPS, containing different concentrations of the aqueous extract (final concentrations ranging from 100 μg/mL to 12.5 μg/mL), was added to the

respective well. The plates were then incubated for an additional period of 24 hours at 37 °C. Negative and positive controls were prepared as described above. The final DMSO concentrations never exceeded 1% v/v.

1.4.3. Biofilm mass quantification

Biofilm biomass was measured by crystal violet staining. Dermatophyte biofilms were stained as described in the original article linked to this supplementary material. Briefly, after removal of the medium, the cells were rinsed with PBS to remove non-adherent cells. The biofilms were then fixed with absolute methanol for 10 min. Next, 100 µl of 0.5% w/v crystal violet solution was applied and the biofilms were allowed to stain for 15 minutes. Once the crystal violet was removed, the biofilms were washed twice with sterile water. The dye was solubilized by adding 150 µl of 33% acetic acid. The solution was then transferred to fresh wells, and the absorbance was measured at 620 nm using a Multiskan™ FC microplate spectrophotometer (Thermo Fisher Scientific Inc., Waltham, Massachusetts, USA). Biomass reduction was calculated using the following equation:

$$\text{Biomass (\%)} = \text{Abs Treatment/Abs CT} \times 100$$

Where Abs CT and Abs Treatment denote the absorbance at 620 nm for control and treated biofilms, respectively.

1.4.4. Biofilm extracellular matrix quantification

The extracellular matrix (ECM) of dermatophyte biofilms was quantified using safranin red as described in the original article linked to this supplementary material. Briefly, after removal of the medium, the biofilms were rinsed with PBS to remove non-adherent cells. Subsequently, 100 µl of 0.5% safranin red solution was added and the biofilms were allowed to stain for 5 min. The solution was then discarded, and the biofilms were washed twice with sterile PBS to remove excess dye. Safranin bound to the biofilm was solubilized with 33% acetic acid. The solution was transferred to fresh wells, and the absorbance was measured at 520 nm using a Multiskan™ FC microplate spectrophotometer (Thermo Fisher Scientific Inc., Waltham, Massachusetts, USA). The reduction in extracellular matrix production was calculated as follows:

$$\text{Extracellular matrix (\%)} = (\text{Abs treatment / Abs CT}) \times 100$$

Where Abs CT and Abs treatment represent the absorbance at 520 nm for control and treated biofilms, respectively.

1.4.5. Biofilm metabolic activity evaluation

The metabolic activity of the biofilms was assessed using the XTT reduction assay (2,3-Bis(2-methoxy-4-nitro-5-sulfophenyl)-2H-tetrazolium-5-carboxanilide) as described in the original article linked to this supplementary material. Briefly, after removal of the culture medium, biofilms were gently washed with PBS to remove any non-adherent cells. An aqueous mixture containing 100 µL of XTT salt (1 mg/mL) and 4 µM menadione (prepared from a 10 mM stock solution in acetone) was added to each well. The plates were then incubated at 37°C for 3 hours. At the end of this period, the absorbance was measured at 490 nm using a Multiskan™ FC microplate spectrophotometer (Thermo Fisher Scientific Inc., Waltham, Massachusetts, USA) to evaluate the metabolic activity of the biofilm, which was calculated using the following equation:

$$\text{Metabolic activity (\%)} = (\text{Abs treatment / Abs CT}) \times 100$$

Where Abs CT and Abs treatment refers to the absorbance values at 490 nm for the control and treated biofilms, respectively.
