# Supplementary material for: Phytochemical Analysis and Therapeutic Potential of Tuberaria lignosa (Sweet) Samp. Aqueous Extract in Skin Injuries
Source: Plants (Basel). 2025 Jul 25;14(15):2299. doi: 10.3390/plants14152299 (PMC12348581; doi:10.3390/plants14152299)

<u>Document S2</u>: Representative chromatograms of punicalagin standard and *Tuberaria lignosa* (Sweet) Samp. aqueous leaf extract from individual analyses by HPLC-DAD for the article "Phytochemical analysis and therapeutic potential of *Tuberaria lignosa* (Sweet) Samp. aqueous extract in skin injuries".

<u>Punicalagin standard (0.8 mg/mL)</u>

Maximum absorbance: 258/378 nm.

Retention time: 4.27 (peak 1) and 4.72 (peak 2).

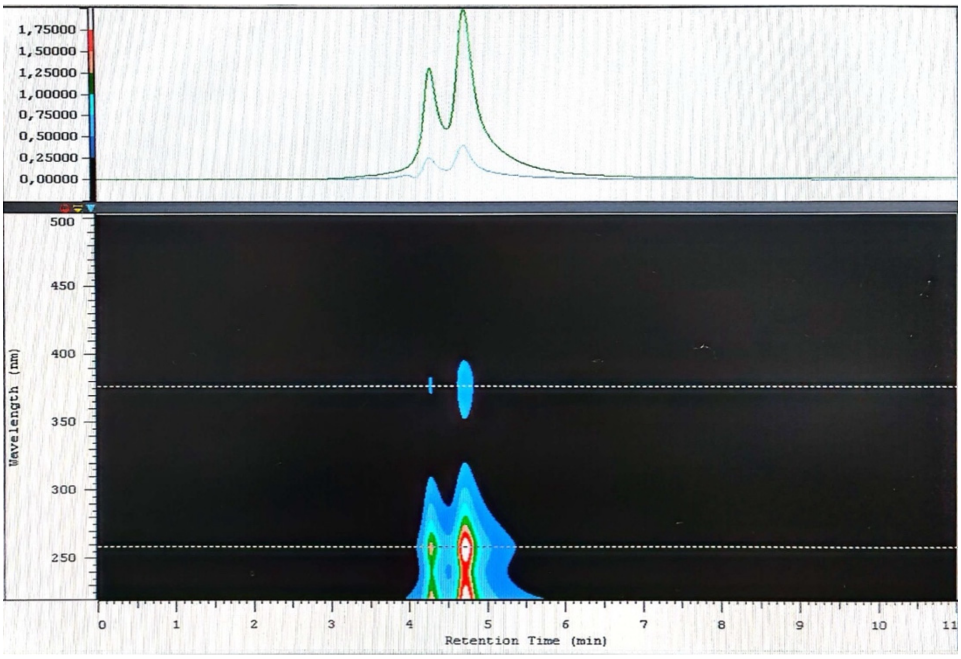

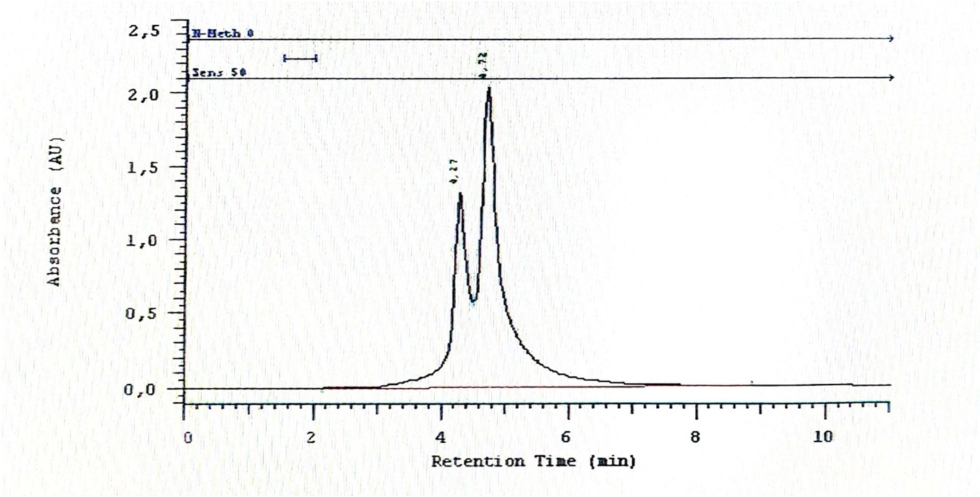

T. lignosa **leaf aqueous extract (10 mg/mL).**

Maximum absorbance: 258/378 nm (Shown slightly above 258 nm for correct scaling)

Retention time: 4.13 (peak 1) and 4.43 (peak 2).

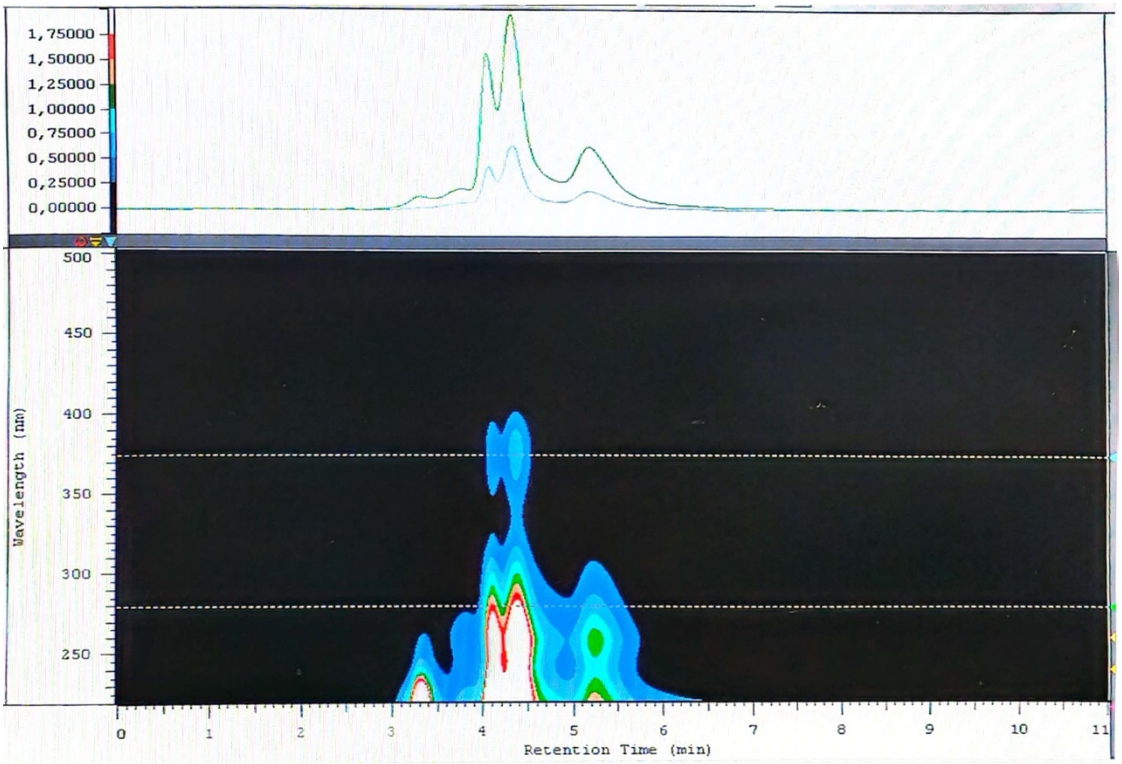

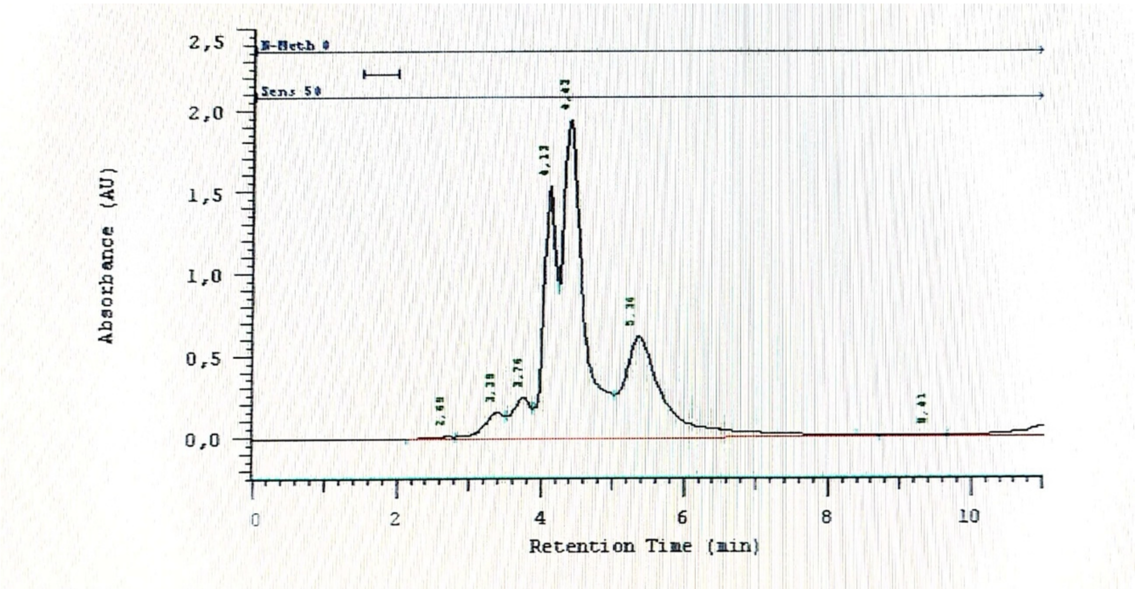

Supplement: Supplementary file 1 [file plants-14-02299-s001.zip › Supplementary Document S2.pdf]
