# Supplementary material for: Phytochemical Analysis and Therapeutic Potential of Tuberaria lignosa (Sweet) Samp. Aqueous Extract in Skin Injuries
Source: Plants (Basel). 2025 Jul 25;14(15):2299. doi: 10.3390/plants14152299 (PMC12348581; doi:10.3390/plants14152299)
Supplement: Supplementary file 1 [file plants-14-02299-s001.zip › Supplementary Table S1.pdf]

Supplementary material

Supplementary Table S1: Calibration curve, correlation coefficient, test range, linear range, LOD and LOQ of phenolic compounds analyzed by UHPLC-HRMS/MS in TLAE.

The limit of detection (LOD) and the limit of quantification (LOQ) were calculated according to the 3σ/10σ approach. In this method, LOD and LOQ are defined as 3.3σ/S and 10σ/S, respectively, where σ is the standard deviation of y-intercepts of the linear regression and S is the slope. The linear range was also established based on the calibration data (Supplementary Table S1). Only the compounds whose concentrations in TLAE were above the calculated LOQ were considered for quantitative analysis.

Supplementary Table S1. Calibration curve, correlation coefficient, test range, linear range, LOD and LOQ of phenolic compounds analyzed by UHPLC-HRMS/MS in TLAE.

Phenolic compound	Calibration curve	Correlation coefficient (R^2)	Test range (μg/mL)	Linear range (μg/mL)	LOD (μg/mL)	LOQ (μg/mL)
Caffeic acid	y = 5.578E+05x - 2.677E+06	0.998	0.01-10	0.01-0.5	0.03	0.09
Hyperoside	y = 1.778E+05x + 8.738E+05	0.997	0.01-10	0.01-1	0.04	0.14
Kaempferol-3,7-di-O-rhamnoside	y = 1.7E+05x – 5.22E+05	0.997	0.01-10	0.01-1	0.03	0.09
Naringenin	y = 1.055E+06x – 7.949E+06	0.996	0.01-10	0.01-0.2	0.02	0.05
Taxifolin	y = 4.546E+05x + 4.402E+06	0.997	0.01-10	0.01-1	0.04	0.12
Vitexin	y = 3.516E+05x – 1.28E+06	0.999	0.01-10	0.1-0.7	0.02	0.06
Isovitexin	y = 3.083E+05x + 1.134E+08	0.997	0.01-10	0.7-10	0.51	1.56
