# Supplementary material for: Phytochemical Analysis and Therapeutic Potential of Tuberaria lignosa (Sweet) Samp. Aqueous Extract in Skin Injuries
Source: Plants (Basel). 2025 Jul 25;14(15):2299. doi: 10.3390/plants14152299 (PMC12348581; doi:10.3390/plants14152299)
Supplement: Supplementary file 1 [file plants-14-02299-s001.zip › Supplementary Table S2.pdf]

Supplementary material

Supplementary Table S2: Calibration curve, correlation coefficient, test range, linear range, LOD and LOQ of punicalagin analyzed by HPLC-DAD in TLAE.

The limit of detection (LOD), limit of quantification (LOQ), calculated according to the 3σ/10σ approach, and linear range were determined (Supplementary Table S2).

Supplementary Table S2. Calibration curve, correlation coefficient, test range, linear range, LOD and LOQ of punicalagin analyzed by HPLC-DAD in TLAE.

Phenolic compound	Calibration curve	Correlation coefficient (R^2)	Test range (mg/mL)	Linear range (mg/mL)	LOD (mg/mL)	LOQ (mg/mL)
Punicalagin	y = 4.53E+07x – 2.67E+06	0.999	0.2-0.8	0.2-0.8	0.07	0.21
